# Pan-genome analysis reveals conservation and diversification of the *SWEET* gene family in wheat

**DOI:** 10.3389/fpls.2026.1949812

**Published:** 2026-10-16

**Authors:** Yanlin Yang, Liuliu Wu, Junyan Sun, Jie Li, Shuhan Liu, Mingming Tang, Zongli Chu, Liping Dong, Shumei Li, Lei Dong

**Affiliations:** 1 College of Agriculture, Xinyang Agriculture and Forestry University, Xinyang, China; 2 School of Pharmacy, Xinyang Agriculture and Forestry University, Xinyang, China; 3 Institute of Biotechnology, Xianghu Laboratory, Hangzhou, China

**Keywords:** abiotic stress, evolutionary diversification, grain traits, pan-genome, presence/absence variation, *SWEET* gene family, wheat

## Abstract

SWEET (Sugars Will Eventually be Exported Transporter) proteins mediate sugar transport and play important roles in plant growth, development, source-sink allocation, and responses to environmental stimuli. However, the conservation and diversification of the *SWEET* gene family across the wheat pan-genome remain poorly understood. Here, we performed a systematic pan-genome analysis of the *SWEET* gene family across 19 hexaploid wheat (*Triticum aestivum* L.) accessions. A total of 1,594 *SWEET* genes were identified, with 77–90 members in individual genomes. Gene copies belonging to core, soft-core, dispensable, and private homologous groups accounted for 52.6%, 30.9%, 14.6%, and 1.9%, respectively, based on presence/absence variation (PAV), revealing a predominantly conserved genomic framework accompanied by a smaller accession-variable component. Phylogenetic, motif, chromosomal and synteny analyses further revealed extensive evolutionary and genomic conservation accompanied by localized diversification among family members. Extensive homeologous and inter-accession collinearity was observed, and most pairwise ortholog comparisons exhibited Ka/Ks ratios below 1, supporting predominant purifying selection on conserved SWEET coding sequences. Promoter analysis identified diverse putative cis-regulatory elements related to environmental, hormonal, and developmental regulation, while transcriptome profiling showed heterogeneous tissue- and stress-associated expression patterns among *TaSWEET* genes. qRT-PCR analysis further suggested distinct temporal responses of selected *TaSWEET* genes under cold and polyethylene glycol (PEG)-induced osmotic stress. *TaSWEET47* overexpression significantly increased grain width, thousand-grain weight, and mature-grain starch content in two independent transgenic lines without significantly affecting grain length. Collectively, these results reveal gene-content, evolutionary, regulatory, and transcriptional diversification within a broadly conserved *SWEET* family framework.

## 1 Introduction

Bread wheat (*Triticum aestivum* L.) is one of the world’s major staple crops, providing a substantial proportion of the calories and protein consumed by humans worldwide (Shiferaw et al., 2013; International Wheat Genome Sequencing Consortium [IWGSC], 2018). Sustaining wheat production is increasingly challenged by environmental stresses and fungal diseases, which can impair photosynthetic carbon assimilation, assimilate transport, reproductive development, and grain filling, ultimately reducing productivity (Thakur et al., 2010; Farooq et al., 2014; Gao et al., 2014). Because grain development depends strongly on the efficient allocation of photoassimilates from source tissues to developing sink organs, sugar transport represents an important component of both yield formation and plant responses to changing environmental conditions (Xu et al., 2022; Fang et al., 2024). Understanding the molecular mechanisms that regulate sugar distribution and source-sink relationships is therefore important for elucidating the genetic basis of wheat development and stress responses.

SWEET (Sugars Will Eventually be Exported Transporter) proteins constitute an evolutionarily conserved family of membrane-localized sugar transporters that mediate facilitated sugar transport across cellular membranes (Chen et al., 2010, 2012; Li et al., 2018). Most plant SWEET proteins contain seven transmembrane helices arranged in a characteristic 3-1–3 architecture comprising two MtN3_slv domains connected by a central transmembrane helix (Tao et al., 2015; Li et al., 2018). Plant SWEET proteins are generally classified into four phylogenetic clades with partially differentiated transport properties and biological functions. Members of Clades I and II predominantly transport hexoses, many Clade III members preferentially transport sucrose and participate in source-sink allocation and reproductive development, whereas Clade IV members are mainly associated with vacuolar sugar transport and intracellular sugar homeostasis (Chen et al., 2010, 2012, 2015b; Chardon et al., 2013; Klemens et al., 2013). For example, *AtSWEET11* and *AtSWEET12* contribute to phloem sucrose export, while *OsSWEET11* and *TaSWEET11* homologs participate in sucrose allocation during seed or grain development (Chen et al., 2012; Ma et al., 2017; Wang et al., 2025). SWEET-mediated sugar transport is also involved in plant-pathogen interactions and responses to abiotic stresses, placing this transporter family at the interface between carbon allocation, development, and environmental responses (Eom et al., 2015; Breia et al., 2021; Gautam et al., 2022).

Bread wheat is an allohexaploid species (AABBDD) with a large and complex genome of approximately 16 Gb, a substantial proportion of which consists of repetitive sequences (IWGSC, 2014, 2018). Its polyploid genome structure, extensive homeologous gene retention, and sequence and structural variation among accessions complicate comprehensive characterization of gene-family diversity (Montenegro et al., 2017; Walkowiak et al., 2020). Although the Chinese Spring (CS) reference genome has provided an essential framework for wheat genomics, a single reference cannot capture all gene-content variation present within bread wheat. The increasing availability of high-quality genome assemblies from diverse wheat accessions has enabled pan-genome approaches that distinguish conserved from variable genomic components and characterize forms of intraspecific variation such as presence/absence variation (PAV), copy-number variation (CNV), and structural variation (SV) (Montenegro et al., 2017; Bayer et al., 2020, 2022; Walkowiak et al., 2020). For example, multi-genome comparison of the wheat nucleotide-binding leucine-rich repeat (NLR) repertoire revealed substantial accession-level variation, demonstrating that analyses based solely on a single reference genome can underestimate gene-family diversity (Walkowiak et al., 2020).

Despite increasing recognition of intraspecific gene-content variation, most *SWEET* family studies have been conducted using single reference genomes. Previous genome-wide analyses in wheat identified *SWEET* family members primarily from the CS reference genome (Gao et al., 2018a; Qin et al., 2020), leaving the extent of *SWEET* gene-content conservation and variation among bread wheat accessions unresolved. Studies in other crops have begun to demonstrate the value of incorporating intraspecific genomic variation into *SWEET*-family analyses. In barley, genome-wide characterization combined with genetic-variation analysis revealed evolutionary and expression divergence among *HvSWEET* genes and provided evidence of artificial selection at *HvSWEET1a* during domestication and improvement (Yue et al., 2023). More recently, pan-genome analysis of the *SWEET* family in *Brassica napus* revealed extensive PAV and integrated gene-content variation with evolutionary and expression analyses (Xu et al., 2026). These studies suggest that a pan-genome framework can reveal *SWEET* diversity that is not apparent from a single reference genome. However, the extent to which the wheat *SWEET* repertoire is conserved or variable across hexaploid wheat accessions, and how such variation relates to evolutionary conservation, regulatory architecture, and transcriptional differentiation, remain poorly understood.

Here, we systematically characterized the *SWEET* gene family across 19 high-quality hexaploid wheat genome assemblies to investigate its conservation and diversification at the pan-genome level. We first defined homologous *SWEET* genes across accessions and quantified their PAV patterns to distinguish conserved and accession-variable components of the family. We then integrated analyses of phylogenetic relationships, conserved motifs, gene structures, chromosomal distribution, syntenic relationships, Ka/Ks-based evolutionary constraints, promoter cis-regulatory elements, and transcriptomic profiles across tissues and under biotic and abiotic stresses. Selected stress-responsive *TaSWEET* genes were further examined by qRT-PCR under cold and PEG-induced osmotic stress. In addition, *TaSWEET47* was functionally evaluated using two independent overexpression lines to assess its contribution to grain-related traits. By integrating pan-genomic variation with evolutionary, regulatory, transcriptional, and functional evidence, this study provides a comprehensive framework for understanding conservation and diversification of the wheat *SWEET* family and identifies candidate genes for further functional investigation.

## 2 Materials and methods

### 2.1 Identification of *SWEET* family members in the wheat pan-genome

Genome assemblies, corresponding gene annotation files and predicted protein sequences of 19 hexaploid bread wheat (*Triticum aestivum* L.; AABBDD) accessions, including CS, Fielder, Abbondanza, Aimengniu, Beijing8, Chuanmai42, Handan6172, Jimai22, Jinmai47, Kuofan11, Mazhamai, Ningchun4, Shi4185, Xinong6028, Xiaoyan6, Yangmai158, Zhoumai16, Zhoumai22, and Zhoumai366, were obtained from publicly available genomic resources, including Ensembl Plants, the KOMUGI database, and the Genome Warehouse (GWH) of the National Genomics Data Center (NGDC). Detailed information on the genome assembly or resource version, assembly accession or project ID, data source, download date, and corresponding reference for each accession is provided in **Supplementary Table 1**.

The publicly released genome assemblies and their corresponding official gene annotations were used without re-annotation. No additional repeat masking was performed in this study. Where repeat-masked assemblies or repeat annotations were provided by the original genome projects, the publicly released resources were used without modification, and the corresponding repeat-masking procedures were those reported in the original genome publications.

To identify *SWEET* family members comprehensively, complementary sequence-similarity- and domain-based approaches were employed. The 17 *Arabidopsis thaliana* (*A. thaliana*) SWEET protein sequences were retrieved from the ePlant database (Waese et al., 2017), and 21 previously characterized *Oryza sativa* (*O. sativa*) SWEET protein sequences were obtained according to Chen et al. (2010) and Yuan et al. (2014). These 38 SWEET proteins were used as reference sequences for homology-based identification. The combined reference protein set was used to search the predicted proteomes of the 19 wheat accessions using BLASTP, with an E-value threshold of < 1 × 10^-5^. To recover potentially divergent SWEET family members, an independent profile hidden Markov model (HMM)-based search was performed against the complete predicted proteome of each wheat accession using the MtN3_slv domain profile (PF03083) from the Pfam database and HMMER v3.1, with an E-value threshold of < 1 × 10^-5^.

Candidate proteins identified by either BLASTP or HMM-based search were combined, and duplicate sequences were removed. The remaining candidates were further examined using Pfam (Mistry et al., 2021) and the NCBI Conserved Domain Database (CDD) to verify the presence and integrity of the MtN3_slv/SWEET domain (Lu et al., 2020). Proteins lacking a recognizable MtN3_slv/SWEET domain or representing clearly incomplete gene models were excluded. The resulting nonredundant set of validated SWEET proteins was retained for subsequent analyses.

Wheat *SWEET* genes were named using accession-specific prefixes to distinguish genes derived from different genome assemblies. Within each accession, the identified *SWEET* genes were ordered according to their original gene-model identifiers and sequentially numbered in ascending order. Genes from CS and Fielder were designated as *TaSWEET1*-*TaSWEETn* and *TaFLDSWEET1*-*TaFLDSWEETn*, respectively, whereas genes from the remaining accessions were assigned corresponding accession-specific prefixes following the same numbering scheme. Thus, the numerical suffixes represent the order of the original gene-model identifiers within each accession and do not indicate orthologous relationships among wheat accessions or correspondence to *SWEET* genes in *A. thaliana* or *O. sativa*.

### 2.2 Physicochemical properties and subcellular localization of SWEET proteins

Physicochemical properties of the identified SWEET proteins, including protein length, molecular weight (MW), theoretical isoelectric point (pI), instability index, aliphatic index, and grand average of hydropathicity (GRAVY), were calculated using TBtools v2.485 (Chen et al., 2023). The presence of an N-terminal methionine was determined by examining the first amino acid of each predicted protein sequence. Proteins showing unusually high or low physicochemical values or lacking an N-terminal methionine were further cross-checked against their original gene annotations and conserved-domain architectures. Subcellular localization was independently predicted using CELLO (Yu et al., 2006) and WoLF PSORT (Horton et al., 2007), and predictions from the two tools were compared for consistency. Physicochemical properties were further compared among the four phylogenetic clades using the Kruskal-Wallis test followed by pairwise comparisons with Bonferroni adjustment. Differences were considered statistically significant at *P* < 0.05.

A TaSWEET47-GFP fusion was constructed in p1300-EGFP and introduced into *Agrobacterium tumefaciens* GV3101 after sequence verification. The fusion protein was co-expressed with the plasma membrane marker SNAP::R. Empty p1300-EGFP (Null::G) co-expressed with Ras1::R, a nuclear and membrane marker, served as the control.

*Agrobacterial* cells were resuspended in an acetosyringone-containing infiltration buffer to an OD_600_ of 0.3. Suspensions carrying the GFP and marker constructs were mixed at 1:1 and infiltrated into tobacco leaves at the four- to eight-leaf stage. After 2 days, bright-field and fluorescence images were acquired using a Leica SP5-X confocal microscope (Leica Microsystems, Germany). Colocalization was assessed from merged GFP and red fluorescence images.

### 2.3 Phylogenetic analysis and classification of SWEET proteins

To investigate the evolutionary relationships of wheat SWEET proteins, the 1,594 validated SWEET protein sequences identified from the 19 wheat accessions were combined with 17 *A. thaliana* and 21 *O. sativa* SWEET protein sequences for phylogenetic analysis. Multiple sequence alignment was performed using ClustalW implemented in MEGA12 with default parameters (Kumar et al., 2024). The best-fitting amino acid substitution model was evaluated in MEGA12 based on the Bayesian information criterion (BIC), and the maximum-likelihood (ML) phylogenetic tree was reconstructed using the LG+G+I model. Evolutionary rate heterogeneity among sites was modeled using a discrete Gamma distribution with five categories (Gamma shape parameter = 1.62), with a proportion of invariant sites of 0.01. Sites with less than 80% coverage were removed using partial deletion. The robustness of the inferred topology was assessed using 1,000 bootstrap replicates.

Wheat SWEET proteins were assigned to the four classical SWEET clades (Clades I-IV) according to their phylogenetic relationships with previously characterized SWEET proteins from *A. thaliana* and *O. sativa* (Chen et al., 2010; Li et al., 2018). The resulting phylogenetic tree was visualized and annotated using iTOL (Letunic and Bork, 2021).

### 2.4 Homologous-locus assignment and PAV classification

To characterize presence/absence variation (PAV) of the *SWEET* gene family across the wheat pan-genome, homologous *SWEET* genes from the 19 wheat accessions were grouped into corresponding homologous groups. An all-versus-all BLASTP analysis was first performed using the 1,594 identified SWEET protein sequences in TBtools v2.485 (Chen et al., 2023). Candidate homologous relationships were retained when pairwise protein sequence identity was >98% and alignment coverage of the longer protein was ≥90% (Guo et al., 2025; Tong et al., 2025). To minimize the erroneous grouping of closely related paralogs, candidate relationships were further evaluated according to their chromosomal locations and subgenomic assignments across accessions. Genes showing high sequence similarity and corresponding chromosomal/subgenomic positions were assigned to the same homologous group, whereas highly similar genes located at non-corresponding genomic positions were treated as separate groups. For the 11 unanchored genes, homologous-group assignment was based on the sequence-similarity criteria because their chromosomal positions were unavailable. These genes were assigned to 10 homologous groups, with the two unanchored CS genes placed in the same group.

For each homologous group, the number of gene copies in each accession was recorded to generate a presence/absence and copy-number matrix. Each homologous group was assigned a PAV category based on its occurrence across the 19 accessions, and genes within the same group inherited the corresponding category (Jiao et al., 2025). Groups present in all 19 accessions were defined as core, those present in 17–18 accessions as soft-core, consistent with the thresholds used by Cheng et al. (2025). Groups present in 2–16 accessions were classified as dispensable, and those detected in only one accession as private.

### 2.5 Conserved motif, domain, and gene-structure analyses

Conserved motifs in the 1,594 SWEET proteins identified across the 19 wheat accessions were analyzed using MEME Suite v5.5.9 (Bailey et al., 2009). Motif discovery was performed in classic mode, with the maximum number of motifs set to 20 and motif widths ranging from 6 to 50 amino acids. Motif similarity was evaluated with Tomtom (Gupta et al., 2007). Pairs with q-values ≤0.05 were checked for alignment overlap, motif length, and sequence profile. Although several pairs showed significant similarity, none were considered redundant, and all 20 motifs were retained. Motif composition and distribution were examined across all validated SWEET proteins. For clade-level comparisons, only proteins assigned to Clades I-IV were included; the two unclassified proteins were retained in the overall motif analysis but excluded from comparisons among phylogenetic clades.

Conserved-domain architectures of all validated SWEET proteins were further characterized using the CDD through Batch CD-Search (Lu et al., 2020). For detailed structural comparison, CS was used as the reference genome, while Fielder was selected because of its amenability to wheat transformation and its chromosome-scale genome assembly (Sato et al., 2021). Gene structures of *SWEET* genes from CS and Fielder were extracted from corresponding GFF3 annotation files. Phylogenetic relationships, conserved motifs, protein domains, and exon-intron structures of the two accessions were integrated and visualized using TBtools v2.485 (Chen et al., 2023).

### 2.6 Chromosomal distribution of *SWEET* genes

Chromosomal positions of *SWEET* genes in CS and Fielder were obtained from their corresponding genome annotation files. Genes assigned to the 21 wheat chromosomes were mapped according to their physical genomic coordinates. Genes annotated on unanchored contigs or sequences with unknown chromosome assignments were retained in the identified *SWEET* gene set but were not included in chromosome-level visualization because definitive chromosome assignments were unavailable in the corresponding genome annotations. The chromosomal distributions of *SWEET* genes across the A, B, and D subgenomes were visualized using MG2C (Chao et al., 2021).

### 2.7 Synteny and collinearity analysis of *SWEET* genes across wheat accessions

For each accession, only the longest protein isoform of each gene was used. Homologous relationships among all 2,493,692 proteins were identified using DIAMOND v2.2.5 in two steps (Buchfink et al., 2021). First, proteins were clustered with diamond linclust with ≥95% amino-acid identity and ≥80% alignment coverage, yielding 198,145 clusters. Then, representative sequences were compared using DIAMOND BLASTP with an E-value cutoff of 1 × 10^-5^ and up to 20 target hits per query. Collinear blocks were detected using MCScanX with MATCH_SCORE = 50, MATCH_SIZE = 5, MAX_GAPS = 25, and OVERLAP_WINDOW = 5, and an E-value cutoff of ≤1 × 10^-5^ (Wang et al., 2012). Each of the 19 genomes was analyzed against itself to identify within-genome collinear blocks, and each of the 18 non-CS accessions was compared directly with CS to identify between-accession collinear blocks. Putative syntenic orthologs were defined as homologous genes from different accessions but the same subgenome located in the same MCScanX collinear blocks.

For two non-CS accessions, syntenic relationships were inferred when genes from the same subgenome shared a CS anchor gene. Collinear homeologs were defined as *SWEET* gene pairs located on different subgenomes (A/B/D) within the same genome and in the same within-genome MCScanX block. Genes on unanchored contigs were excluded from chromosome-level analyses. For visualization, the 21 chromosomes (1A-7D) of each accession were arranged in order with chromosome boundaries retained, resulting in 19 accession sectors and 399 chromosome segments. The circular plot was generated using the R package circlize (Gu et al., 2014).

### 2.8 Ka/Ks analysis of orthologous *SWEET* gene pairs

Coding sequences (CDSs) of *SWEET* genes were extracted from the 19 wheat accessions. Corresponding *SWEET* genes across accessions were assigned to the same gene locus based on their established orthologous relationships, and all possible inter-accession gene pairs within each locus were compared. For each gene pair, CDSs were translated into amino acid sequences and globally aligned at the protein level. The resulting protein alignments were then back-translated to codon alignments based on ungapped matched positions.

Nonsynonymous substitution rates (Ka), synonymous substitution rates (Ks), and Ka/Ks ratios were estimated using the Nei-Gojobori method (Nei and Gojobori, 1986), corresponding to the NG algorithm implemented in KaKs_Calculator 3.0 (Zhang, 2022). To improve the reliability of Ka/Ks estimates, gene pairs with Ks < 0.001 were excluded to avoid inflated ratios caused by near-zero denominators, whereas pairs with Ks > 1.5 were removed to reduce the potential influence of substitution saturation. Ka/Ks ratios < 1, = 1, and > 1 were interpreted as being consistent with purifying selection, neutral evolution, and potential positive selection, respectively. Ka/Ks = 1 represents the neutral expectation. Ka/Ks distributions were visualized using violin plots, with Ka/Ks = 1 used as the reference threshold.

### 2.9 Comparative synteny analysis among wheat, rice, and *Arabidopsis*

Comparative synteny analysis was performed to investigate the evolutionary conservation of *SWEET* genes between CS wheat and *O. sativa*, and between CS wheat and *A. thaliana*. The CS genome was used to represent bread wheat in the interspecific comparison. Genome annotation files and corresponding protein sequences for rice and *A. thaliana* were downloaded from Ensembl Plants. Pairwise protein sequence similarity searches were performed using BLASTP with an E-value threshold of 1 × 10^-5^, and the resulting similarity information together with genomic coordinates was used for synteny analysis with MCScanX (Wang et al., 2012). Syntenic relationships involving *SWEET* genes were extracted from the resulting collinear blocks, and comparative synteny maps were generated using TBtools v2.485 (Chen et al., 2023).

### 2.10 Cis-regulatory element analysis

For each *SWEET* gene, the 2-kb genomic sequence upstream of the translation start site was extracted from the corresponding wheat genome assembly and used for cis-regulatory element prediction. Cis-regulatory elements within these promoter sequences were predicted using PlantCARE (Lescot et al., 2002). Core promoter elements, including TATA-box and CAAT-box motifs, as well as elements with ambiguous or unclassified functional annotations, were excluded from downstream comparative analysis. The retained cis-regulatory elements were grouped according to their annotated functions into three broad categories: environmental- and light-responsive, phytohormone-responsive, and growth- and development-related elements. The occurrence of each retained cis-regulatory element was summarized across the identified *SWEET* genes and visualized using TBtools v2.485 (Chen et al., 2023).

### 2.11 Transcriptome expression analysis of *TaSWEET* genes

Publicly available RNA-seq datasets were obtained from WheatOmics 1.0 (Ma et al., 2021) to examine the expression patterns of *TaSWEET* genes across different tissues and in response to biotic and abiotic stresses. The tissue and developmental-stage dataset was derived from CS and included roots, stems, leaves, spikes, and grains. The biotic-stress dataset was derived from the wheat line N9134 following infection with powdery mildew (*Blumeria graminis* f. sp. *tritici*, *Bgt*) and stripe rust (*Puccinia striiformis* f. sp. *tritici*, *Pst*). The abiotic-stress datasets included drought and heat treatments of TAM107 and cold treatment of Manitou. The developmental expression profile of *TaSWEET47* (*TraesCS6A02G218800*) was further examined using publicly available carpel RNA-seq data from Longchun35, covering 12 stages from the green anther stage to 35 days post-anthesis (Liu et al., 2025). TPM values were used to plot the expression profile without Z-score transformation. Detailed information on the accessions, treatments, sampling time points, original project accessions, and data sources is provided in **Supplementary Table 11B**.

Transcript abundance values were used to examine the relative expression patterns of *TaSWEET* genes across tissues and treatments. For heatmap visualization, expression values were standardized for each gene across the corresponding samples using row-wise Z-score normalization, and heatmaps were generated using TBtools v2.485 (Chen et al., 2023). Because these analyses were intended to characterize relative expression patterns rather than to perform formal differential-expression analysis, genes were not classified as differentially expressed on the basis of fold-change or statistical-significance thresholds. The expression values used for visualization are provided in **Supplementary Table 11A**. Six candidate *TaSWEET* genes showing divergent expression trends under cold and drought were selected for subsequent qRT-PCR analysis.

To integrate PAV, cis-element, phylogenetic, and tissue-expression results, a focused analysis was performed for CS TaSWEET genes (**Supplementary Table 12**). Because comparable tissue-expression data were only available for CS, this analysis was restricted to CS genes. PAV categories and phylogenetic clades were obtained from the previous analyses. ABRE, MBS, DRE, and LTR elements in each 2-kb upstream region were summed as drought- and cold-related cis-elements. Non-private genes included core, soft-core, and dispensable genes.

Mean TPM values were calculated for root, stem, leaf, spike, and grain without Z-score transformation. The highest mean TPM among the five tissues was defined as the maximum tissue TPM. Genes with a maximum tissue TPM ≥ 1 were considered expressed (Cheng et al., 2017). Among these, genes with the highest mean TPM in grain were classified as grain-preferential (Nguyen et al., 2026).

Statistical analyses were performed using IBM SPSS Statistics 27.0 (IBM Corp., Armonk, NY, USA). Total cis-element number and maximum tissue TPM were compared between private and non-private genes using two-sided Mann-Whitney U tests. The proportions of core and grain-preferential genes were compared between Clade III and the other clades using two-sided Fisher’s exact tests. Continuous data are presented as median (Q1-Q3), and P < 0.05 was considered significant.

### 2.12 Plant materials, stress treatments, and qRT-PCR analysis

CS seedlings were used for qRT-PCR to examine the expression of the six *TaSWEET* genes identified in the transcriptome analysis in Section 2.11 under cold and PEG-induced osmotic stress. For cold treatment, plants were exposed to 4°C, whereas osmotic stress was imposed using 20% (w/v) PEG 6000. Samples were collected at 0, 6, 12, 24, and 48 h after treatment. Leaf samples were collected at each time point, immediately frozen in liquid nitrogen, and stored at −80°C until RNA extraction. Three independent biological replicates were collected for each treatment and time point.

Total RNA was extracted using a DP430 RNA Extraction Kit (Tiangen Biotech, Beijing, China) according to the manufacturer’s instructions. RNA concentration and purity were measured using a NanoDrop spectrophotometer, and RNA integrity was assessed by 1% agarose gel electrophoresis. First-strand cDNA was synthesized from 1 μg of total RNA using the HiScript 1st Strand cDNA Synthesis Kit (Vazyme, Nanjing, China), and qRT-PCR was performed using an ABI QuantStudio Real-Time PCR System (Applied Biosystems, USA) with AceQ SYBR Green Master Mix (Vazyme, Nanjing, China). Each reaction contained 2 μL of cDNA. *TaACTIN* and *TaGAPDH* were independently used as reference genes to evaluate the robustness of the expression patterns. Relative transcript abundance was calculated using the 2^-ΔΔCt^ method, with the 0 h samples as the baseline (Livak and Schmittgen, 2001). The same *TaACTIN* primer pair was previously used for qRT-PCR analysis in wheat under drought stress (Erkılıç et al., 2024), whereas the *TaGAPDH* primer pair was the same as that used by Gao et al. (2018b). Three independent biological replicates were analyzed for each sample. Gene-specific primers used for qRT-PCR are listed in **Supplementary Table 16A**. Melting curve analysis was performed after amplification, and a single peak was observed for each primer pair. Representative melting curves for the six target genes and the two reference genes, *TaACTIN* and *TaGAPDH*, are provided in **Supplementary Figure 1**. Ct values from the three technical replicates were averaged for each biological replicate.

### 2.13 Construction and characterization of *TaSWEET47* overexpression lines

The full-length coding sequence (CDS) of *TaSWEET47* was cloned into the pLGY-OE3 overexpression vector under the control of the maize ubiquitin (Ubi) promoter. The pLGY-OE3 vector was provided by Prof. Genying Li’s laboratory at the Shandong Academy of Agricultural Sciences. The resulting *TaSWEET47* overexpression construct was generated in our laboratory and subsequently submitted to Wuhan Boyuan Biotechnology Co., Ltd. for genetic transformation of the wheat cultivar Fielder. Regenerated plants obtained from the transformation service were subsequently screened and characterized in our laboratory.

Putative transgenic plants were identified by PCR using transgene-specific primers, and *TaSWEET47* transcript abundance was examined by qRT-PCR using *TaACTIN* as the reference gene. Two independent T2 overexpression lines with confirmed positive amplification and elevated *TaSWEET47* expression were selected for phenotypic analysis. At maturity, grains were harvested from Fielder and the two independent overexpression lines, and thousand-grain weight, grain width, and grain length were measured. Primers used for transgenic-plant identification and expression analysis are provided in **Supplementary Table 16B**.

### 2.14 Determination of grain starch content

Total starch content was measured in mature grains of Fielder and two independent *TaSWEET47*-overexpression lines (OE-1 and OE-2) following the Chinese national standard GB 5009.9-2023 (National Health Commission of the People’s Republic of China and State Administration for Market Regulation, 2023). Grain samples were ground and passed through a 0.425-mm sieve. Soluble sugars were removed before starch hydrolysis, and the released reducing sugars were measured using alkaline copper tartrate solution. Starch content was calculated according to the standard method and expressed as g/100 g of sample. Three biological replicates were analyzed for each genotype.

### 2.15 Statistical analysis

Statistical analyses were performed using SPSS 27.0 (IBM Corp., Armonk, NY, USA). Unless otherwise stated, data are presented as the mean ± standard deviation (SD) of three independent biological replicates. Differences between two groups were evaluated using Student’s t-test, whereas comparisons involving multiple groups were performed using one-way analysis of variance (ANOVA) followed by the least significant difference (LSD) test or Duncan’s multiple range test, as appropriate. For data that did not meet the assumptions of parametric analysis, differences among groups were evaluated using the Kruskal-Wallis test followed by pairwise comparisons with Bonferroni adjustment. Statistical significance was defined as *P* < 0.05. Different lowercase letters or asterisks in the figures indicate statistically significant differences as specified in the corresponding figure legends.

## 3 Results

### 3.1 Identification and PAV-based classification of the wheat *SWEET* gene family

Using complementary homology- and domain-based searches, a total of 1,594 *SWEET* genes were identified across the 19 hexaploid wheat accessions, with 77–90 members detected in individual genomes (**Figure 1A**; **Supplementary Table 2**). The number of *SWEET* genes varied moderately among accessions, indicating variation in family size across the wheat pan-genome.

**Figure 1:**
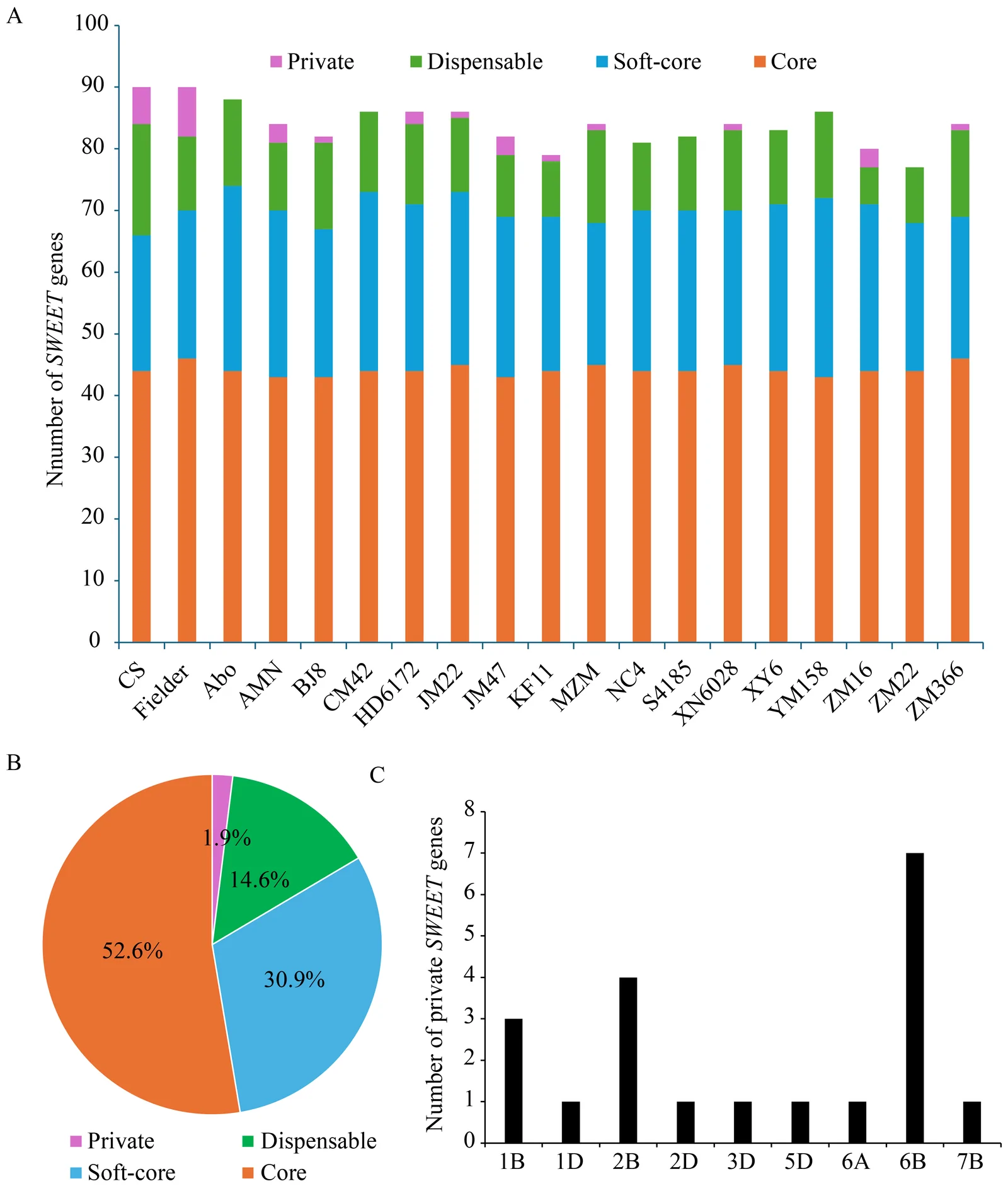
Pan-genome composition and distribution of the wheat *SWEET* gene family across 19 hexaploid wheat accessions. **(A)** Numbers of *SWEET* genes identified in each wheat accession, with genes grouped according to the PAV categories of their corresponding homologous loci. **(B)** Proportions of the 1,594 *SWEET* gene copies belonging to the core, soft-core, dispensable, and private PAV categories according to the occurrence frequencies of their corresponding homologous groups across the 19 accessions. Core groups were detected in all 19 accessions, soft-core groups in 17 -18 accessions, dispensable groups in 2 -16 accessions, and private groups in a single accession. **(C)** Chromosomal distribution of private *SWEET* genes across the 19 wheat accessions.

To facilitate gene tracking across different genome assemblies, the identified *SWEET* genes were assigned accession-specific prefixes and sequentially numbered according to the order of their original gene-model identifiers within each accession. The numerical suffixes therefore serve as accession-specific identifiers and do not indicate orthologous relationships among wheat accessions or correspondence to *SWEET* genes from *A. thaliana* or *O. sativa* (**Supplementary Table 2**).

To assess gene-content conservation and variation across the wheat pan-genome, the 1,594 wheat *SWEET* genes were organized into homologous groups based on protein sequence similarity together with corresponding chromosomal/subgenomic positions. Each homologous group was assigned a PAV category based on its occurrence across the 19 accessions, and its constituent genes inherited the corresponding category. Of the 1,594 gene copies, 52.6% of the gene copies were classified as core, 30.9% as soft-core, 14.6% as dispensable, and 1.9% as private (**Figure 1B**; **Supplementary Table 3**). Core and soft-core gene copies together accounted for 83.5% of the *SWEET* pan-genome, whereas dispensable and private groups accounted for the remaining 16.5%.

Private *SWEET* groups were distributed across 9 chromosomes, with the largest number detected on chromosome 6B (seven groups), followed by chromosomes 2B (four groups) and 1B (three groups) (**Figure 1C**). Collectively, the predominance of core and soft-core groups indicates that most *SWEET* homologous groups are widely conserved across the 19 wheat accessions, whereas a smaller proportion exhibits presence/absence variation (**Supplementary Table 4**).

To further examine whether the PAV composition of the *SWEET* family varied among wheat cultivars released during different breeding periods, 17 Chinese accessions were grouped into three breeding periods: the 1950s-1960s, the 1980s-1990s, and the post-2000s period (**Supplementary Tables 5A, B**). The total number of *SWEET* genes remained relatively stable among the three groups, with mean values of 83.33, 83.71, and 82.57 genes, respectively, and no significant difference was detected among breeding periods (Kruskal-Wallis test, *P* = 0.949). Similarly, the numbers of core, soft-core, dispensable, private, and variable *SWEET* genes did not differ significantly among the three breeding periods (*P* = 0.114, 0.074, 0.124, 0.513, and 0.105, respectively) (**Supplementary Table 5C**). Overall, these results indicate that the composition of the *SWEET* gene family was highly stable across the three breeding periods examined.

### 3.2 Physicochemical properties and predicted subcellular localization of wheat SWEET proteins

The 1,594 SWEET proteins identified across the 19 wheat accessions exhibited substantial variation in their physicochemical properties (**Supplementary Table 6A**). Molecular weight (MW) ranged from 24.62 to 43.52 kDa, theoretical isoelectric point (pI) from 5.40 to 10.27, instability index from 27.66 to 59.29, aliphatic index from 95.06 to 126.29, and grand average of hydropathicity (GRAVY) from 0.320 to 0.872. Notably, all GRAVY values were positive, indicating an overall hydrophobic character of the identified SWEET proteins. Five predicted SWEET proteins lacked an annotated N-terminal methionine. These proteins were retained after cross-checking their original gene annotations, protein sequences, and conserved-domain architectures.

Subcellular localization predictions also showed a strong predominance of plasma-membrane localization (**Supplementary Table 6A**). By CELLO, all 1,594 SWEET proteins were predicted to localize to the plasma membrane. In contrast, WoLF PSORT assigned 1,314 proteins to the plasma membrane, 207 to the vacuolar membrane, 71 to chloroplasts, and two to the endoplasmic reticulum. Consequently, the two prediction methods produced consistent plasma-membrane assignments for 1,314 proteins (82.4%), whereas 280 proteins (17.6%) showed discordant predictions. Overall, the two prediction methods supported predominant plasma-membrane localization of wheat SWEET proteins, while also revealing prediction uncertainty for a subset of family members.

To examine the subcellular localization of TaSWEET47, a GFP fusion protein was co-expressed with the plasma membrane marker SNAP::R. The GFP fluorescence was mainly observed at the cell periphery and overlapped with the marker signal, supporting predominant plasma membrane localization of TaSWEET47 (**Supplementary Figure 2**).

### 3.3 Phylogenetic relationships and structural characteristics of wheat SWEET proteins

Phylogenetic analysis based on 1,594 wheat SWEET proteins together with 17 *A. thaliana* and 21 *O. sativa* SWEET reference proteins recovered the four major clades previously recognized in the SWEET family (**Figure 2**). Of the 1,594 wheat SWEET proteins, 1,592 were assigned to the four reference-defined clades, including 277, 593, 599 and 123 proteins in Clades I, II, III, and IV, respectively. Clade III contained the largest number of assigned wheat SWEET proteins, closely followed by Clade II, whereas Clade IV contained the fewest members. Two highly divergent proteins, *TaZM366SWEET31* and *TaXN6028SWEET14*, showed ambiguous phylogenetic positions and were retained as unclassified. Wheat SWEET proteins from different accessions frequently clustered together, indicating broad conservation of corresponding family members across the wheat pan-genome.

**Figure 2:**
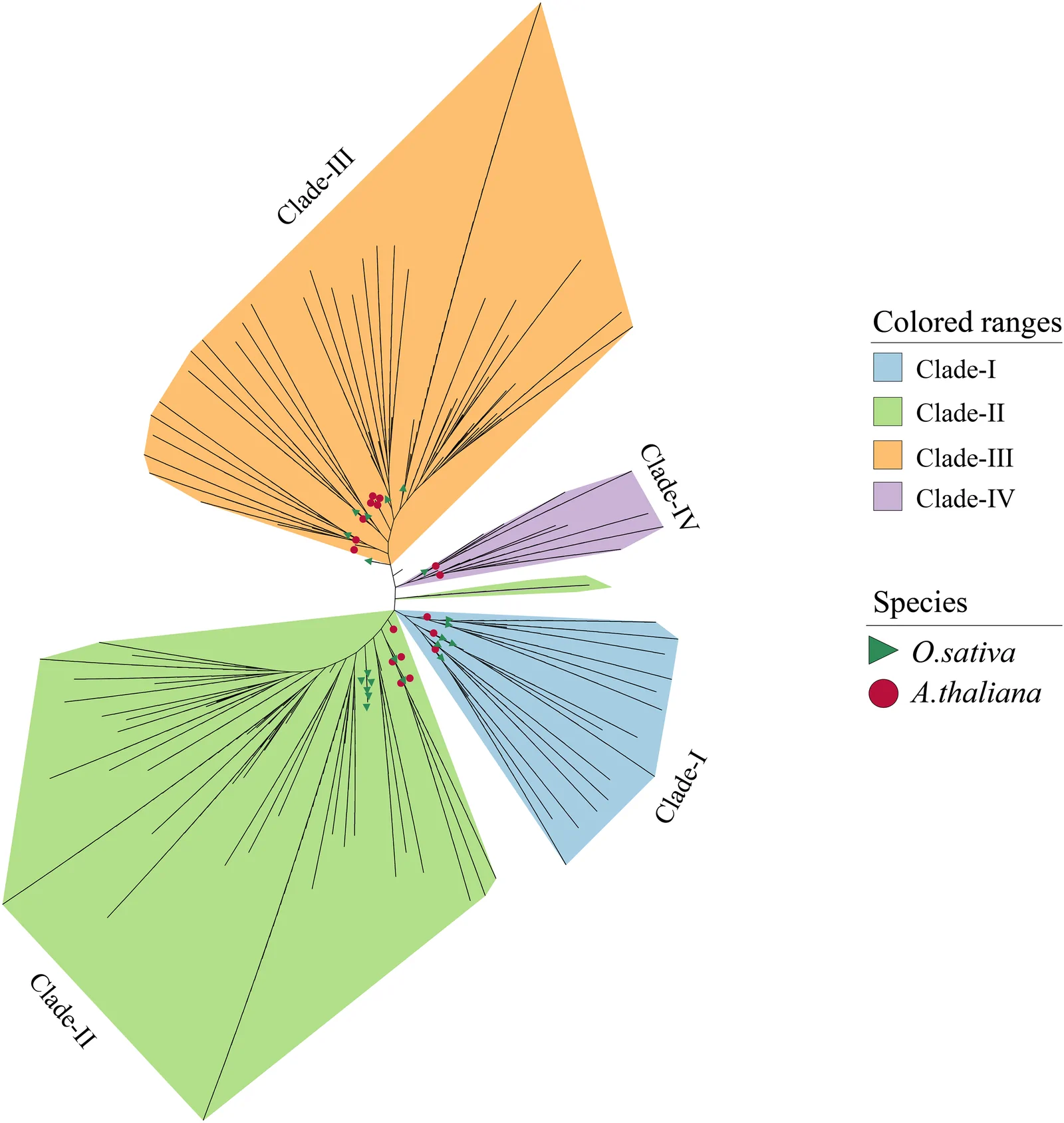
Maximum-likelihood phylogenetic analysis of SWEET proteins from 19 wheat accessions, *A. thaliana*, and *O. sativa*. A total of 1,594 TaSWEET proteins, 17 *A. thaliana SWEET* proteins, and 21 *O. sativa* SWEET proteins were aligned using ClustalW in MEGA12 with default parameters. The maximum-likelihood tree was constructed using the LG+G+I model with five gamma categories, a gamma shape parameter of 1.62, and an invariant-site proportion of 0.01. Gaps and missing data were treated by partial deletion with an 80% site coverage cutoff. Branch support was assessed with 1,000 bootstrap replicates. SWEET proteins were divided into four clades (Clades I-IV) based on their phylogenetic relationships with *A. thaliana* and *O. sativa* reference proteins. Colored ranges show the four clades. Red circles and green triangles indicate *A. thaliana* and *O. sativa* SWEET proteins, respectively. The tree was visualized and annotated in iTOL.

Among the 1,592 proteins assigned to the four reference-defined phylogenetic clades, physicochemical properties differed significantly among Clades I-IV (**Supplementary Table 6B**). Significant differences were detected for MW, pI, instability index, aliphatic index, and GRAVY (Kruskal-Wallis test, *P* < 0.001 for all five parameters). Clade III proteins showed the highest median MW (32.28 kDa) and instability index (40.05), whereas Clade II proteins had the highest median pI (9.28) and aliphatic index (121.99). GRAVY values also differed among the four clades, with Bonferroni adjusted pairwise comparisons showing significant differences between Clade IV and Clades II and III, whereas Clade I did not differ significantly from the other clades. These results indicate significant differences in several physicochemical properties among the four phylogenetic clades.

Conserved-motif analysis of all 1,594 wheat SWEET proteins identified 20 motifs (**Supplementary Table 7**). Motifs 1, 2, 3, and 5 were highly frequent, occurring in 96.8-99.9% of the proteins, whereas Motifs 16 and 19 occurred in only 3.1% and 7.2% (**Supplementary Table 7B**), respectively. Among the 1,592 proteins assigned to the four classical phylogenetic clades, motif distribution varied among clades (**Supplementary Tables 7E, F**). Motifs 16 and 19 were restricted to Clade I, Motifs 11 and 20 to Clade II, and Motifs 12 and 17 to Clade III. Motif 6 was present in Clades III and IV but absent from Clades I and II (**Supplementary Tables 7E, F**). The average number of motifs per protein was 6.62, 9.48, 10.28, and 7.64 in Clades I-IV, respectively, with the highest value in Clade III and the lowest in Clade I (**Supplementary Table 7E**). Motif composition was broadly conserved across the 19 wheat accessions, although the frequencies of individual motifs showed some variation among accessions (**Supplementary Tables 7C, D**).

Detailed structural analyses were carried out separately for CS and Fielder by combining phylogenetic relationships, conserved motifs, protein domains, and gene structures (**Figure 3**). In both accessions, SWEET proteins were grouped into four phylogenetic clades. Proteins within the same clade generally had similar motif compositions and arrangements, whereas motif number and organization varied among clades (**Figures 3A, D**). Motif 1 was highly conserved in both accessions. Consistent with the identification criteria, all SWEET proteins in CS and Fielder contained two MtN3_slv-related domains (**Figures 3B, E**). Gene-structure analysis showed that *SWEET* genes contained four to seven exons in both accessions, and genes within the same phylogenetic clade generally had similar exon-intron structures (**Figures 3C, F**). Overall, motif composition, domain architecture, and gene structure were largely conserved within clades, while differences were observed among clades.

**Figure 3:**
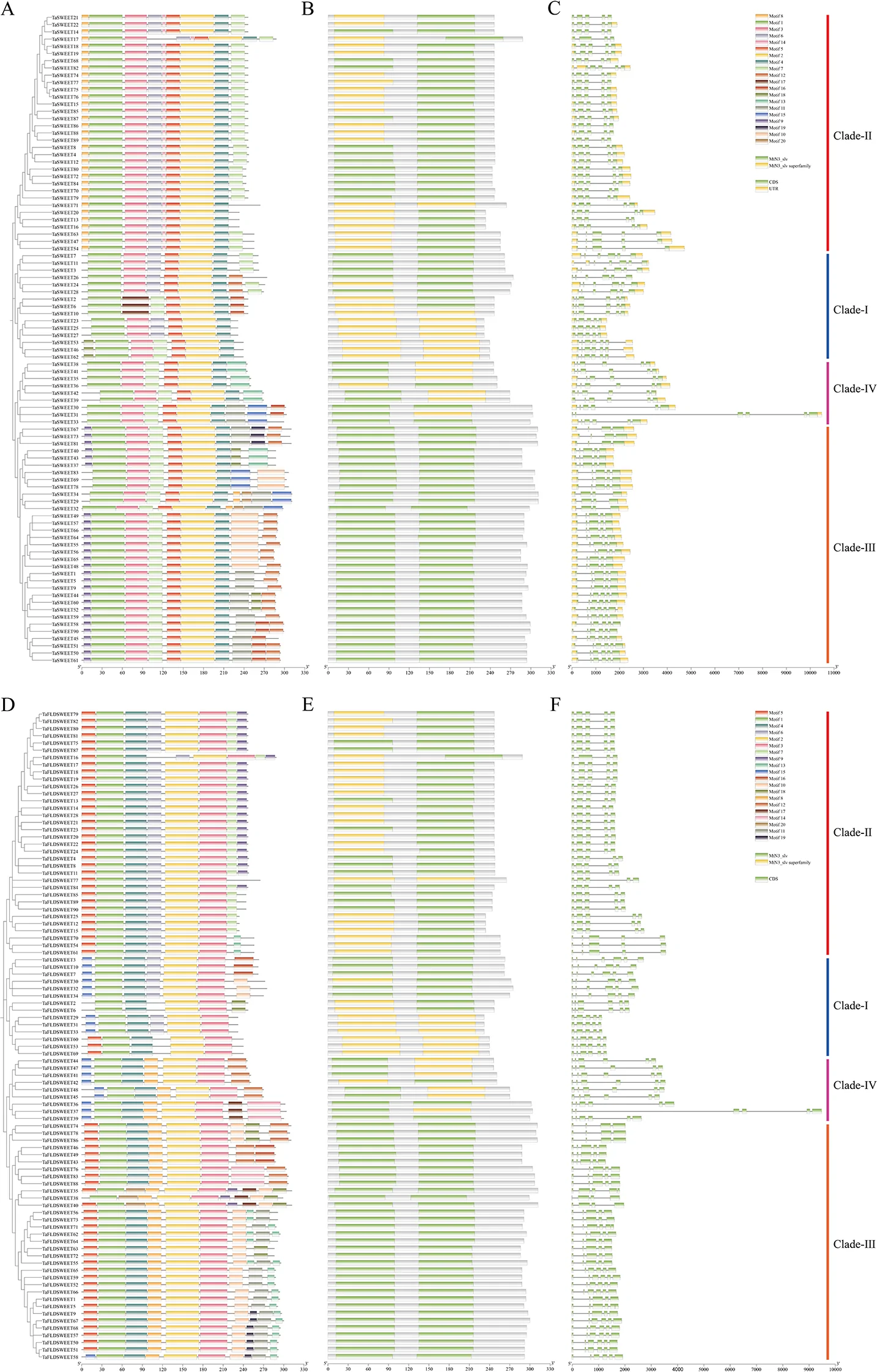
Conserved motifs, domain architectures, and gene structures of *SWEET* family members in Chinese Spring and Fielder. **(A–C)** Structural characteristics of SWEET family members in Chinese Spring. **(D–F)** Structural characteristics of *SWEET* family members in Fielder. **(A, D)** Distribution of 20 conserved motifs identified by MEME, with different colored boxes representing individual motifs. **(B, E)** Distribution of MtN3_slv-related conserved domains. Green and yellow boxes represent MtN3_slv and MtN3_slv superfamily domains, respectively. **(C, F)** Exon-intron structures of *SWEET* genes, with green and yellow boxes representing coding sequences (CDSs) and untranslated regions (UTRs), respectively. Phylogenetic clade assignments (Clades I-IV) are indicated on the right.

### 3.4 Chromosomal distribution of *TaSWEET* genes

The chromosomal distributions of *SWEET* genes were examined in CS and Fielder (**Figure 4**; **Supplementary Table 8A**). In CS, 84 of the 90 identified *SWEET* genes were anchored to the 21 chromosomes, including 26, 31, and 27 genes on the A, B, and D subgenomes, respectively. In Fielder, 89 of the 90 identified *SWEET* genes were chromosome-anchored, with 25, 37, and 27 genes located on the A, B, and D subgenomes, respectively. Thus, the B subgenome contained the largest number of chromosome-anchored *SWEET* genes in both accessions.

**Figure 4:**
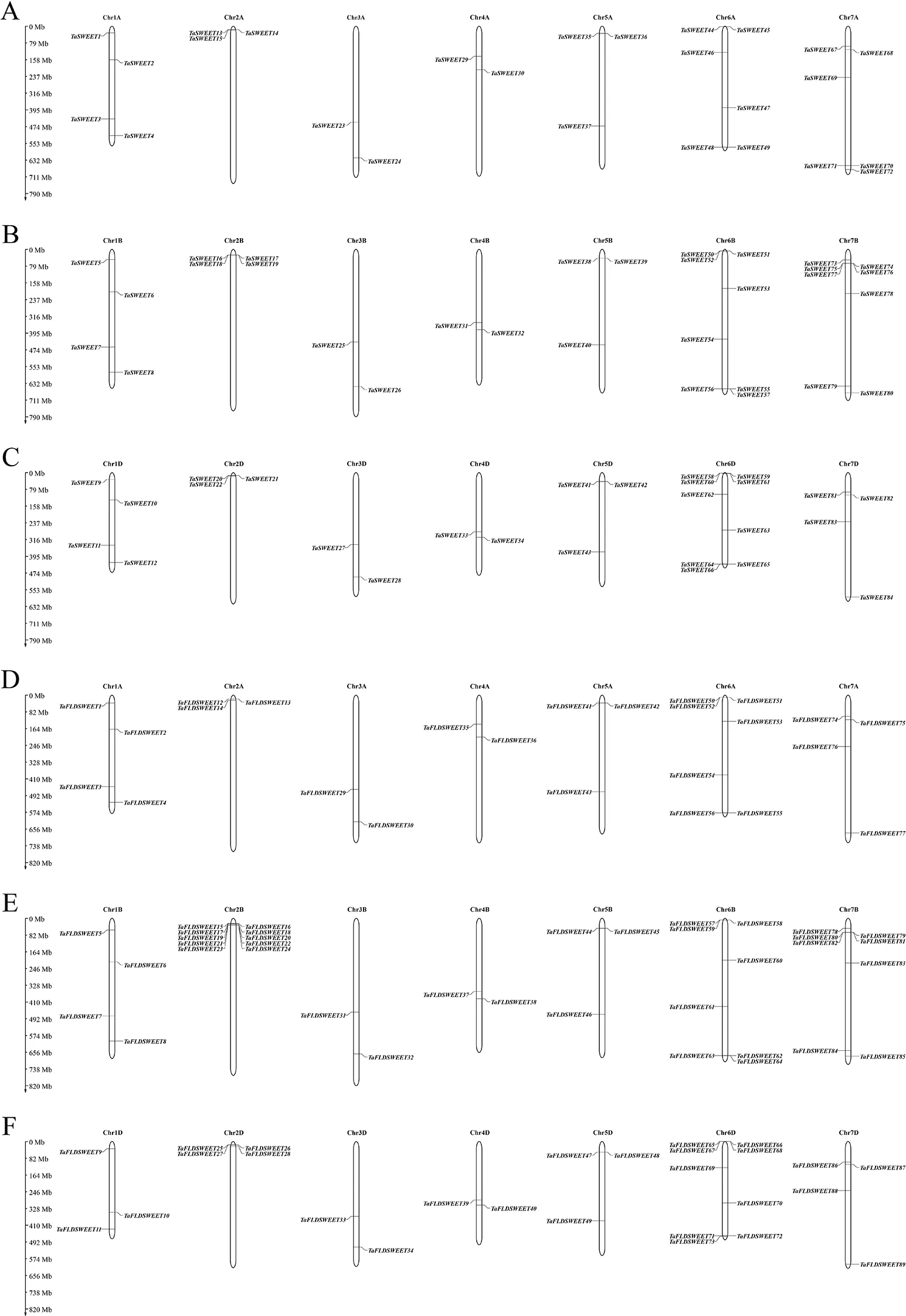
Chromosomal distribution of *SWEET* genes in CS and Fielder. The physical positions of *SWEET* genes are shown on the 21 chromosomes of CS **(A–C)** and Fielder **(D–F)**. **(A, D)** A-subgenome chromosomes; **(B, E)** B-subgenome chromosomes; and **(C, F)** D-subgenome chromosomes. Chromosome numbers are indicated above each chromosome, and physical positions are shown in megabases (Mb). Gene names are displayed adjacent to their corresponding chromosomal positions.

The chromosome-anchored *SWEET* genes were unevenly distributed across the 21 chromosomes in both CS and Fielder (**Figure 4**). Although the overall distribution patterns were broadly comparable between the two accessions, variation in gene number was observed among individual chromosomes and subgenomes. Of the 180 *SWEET* genes identified in the two accessions, seven were not included in the chromosome-level maps: six in CS and one in Fielder. These genes were retained in the identified *SWEET* gene sets but were not displayed in **Figure 4** because their corresponding gene models were assigned to unanchored or non-standard genomic sequences rather than to one of the 21 standard chromosomes in the original genome annotations.

Overall, *SWEET* genes showed a broadly similar but uneven chromosomal distribution in CS and Fielder, with the B subgenome harboring the largest number of chromosome-anchored members in both accessions.

### 3.5 Synteny and conservation of *SWEET* genes across wheat accessions

Comparative synteny analysis revealed extensive collinear relationships among *SWEET* genes across the 19 wheat accessions (**Figure 5**; **Supplementary Table 8**). Of the 1,594 *SWEET* genes, 1,583 were anchored to A, B, or D chromosomes and included in the analysis, while 11 genes on unanchored contigs were excluded (**Supplementary Table 8A**). Across the 171 cultivar pairs, 11,885 putative syntenic orthologous relationships were identified. The number ranged from 62 to 75 per accession pair, with a mean of 69.5 and a median of 70 (**Figure 5B**; **Supplementary Table 8B**). The highest number of syntenic ortholog pairs (75) was observed between CS and Fielder, Abbondanza, Handan6172, or Jimai22, whereas the lowest number (62) occurred between Zhoumai16 and Zhoumai366 and between Zhoumai22 and Zhoumai366. Among the other 18 accessions, 55–60 *SWEET* genes per accession were involved in syntenic relationships with CS (**Supplementary Table 8A**). Within individual genomes, 1,219 homeologous collinear pairs were identified, including 417 A-B, 379 A-D, and 423 B-D pairs (**Supplementary Table 8C**). Overall, 1,071 of the 1,583 chromosome-anchored *SWEET* genes (67.7%) were involved in homeologous collinearity. The pooled proportions were 72.4% in the A subgenome, 63.0% in B, and 68.5% in D, although the patterns varied among accessions (**Supplementary Table 8C**). Corresponding syntenic *SWEET* genes generally retained conserved chromosome and subgenome relationships across accessions, indicating substantial conservation of their genomic organization within the wheat pan-genome.

**Figure 5:**
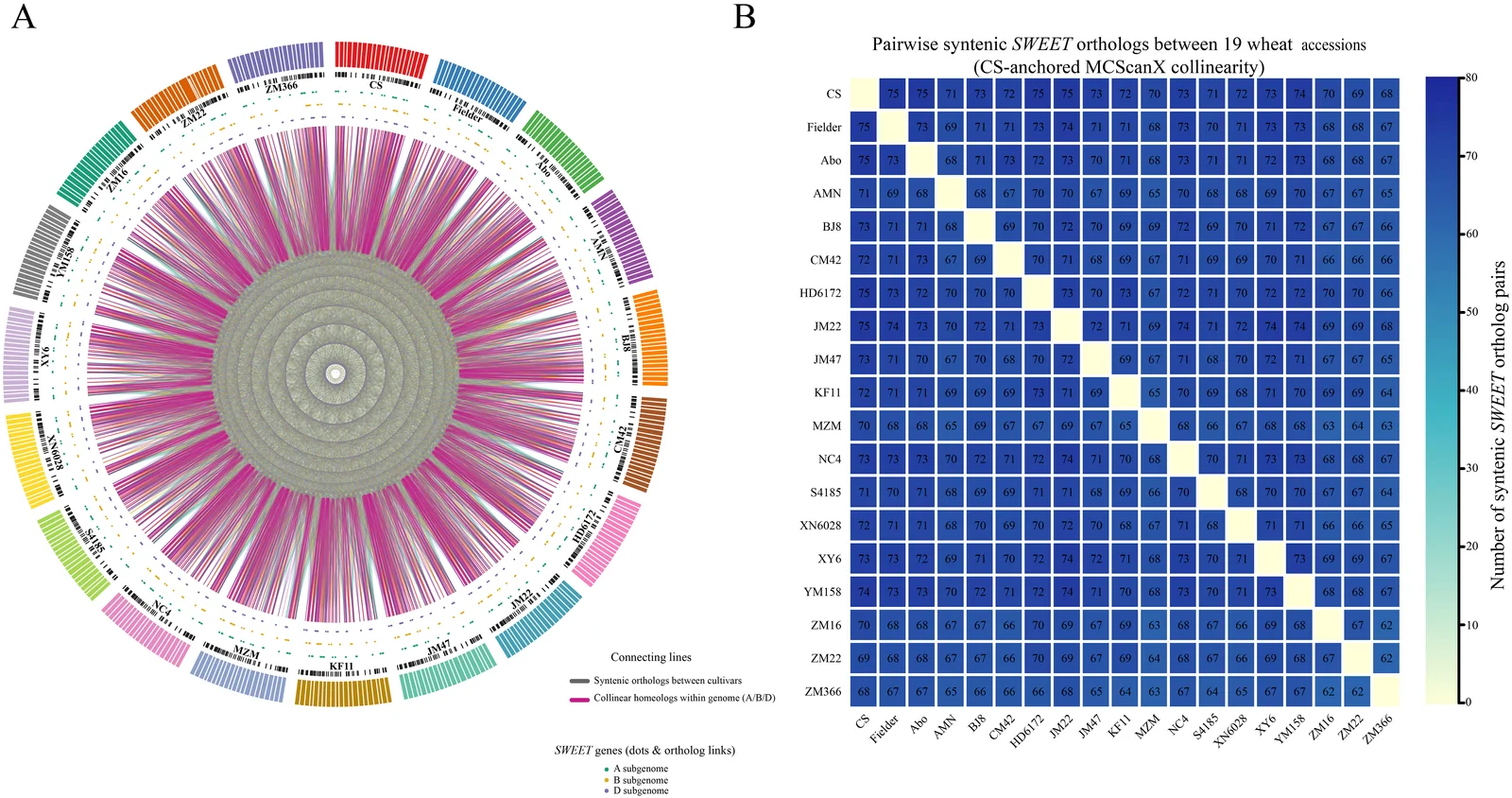
Collinearity and syntenic relationships of *SWEET* genes across 19 hexaploid wheat accessions. **(A)** Genome-wide visualization of *SWEET* syntenic and collinear relationships across the 19 wheat accessions. Colored dots indicate *SWEET* genes located on the A, B, and D subgenomes. Connecting lines represent syntenic orthologous relationships between accessions and homeologous collinear relationships among the A, B, and D subgenomes within individual genomes, as indicated in the legend. **(B)** Pairwise numbers of syntenic *SWEET* orthologs among the 19 wheat accessions based on CS-anchored MCScanX collinearity analysis. Numbers within cells indicate the number of syntenic *SWEET* ortholog pairs identified for each pairwise accession comparison.

Nevertheless, local variation in syntenic relationships was observed among the wheat genomes. Some *SWEET* genes lacked detectable collinear counterparts in one or more pairwise comparisons, indicating variation in the conservation of individual genomic regions among accessions. These differences were broadly consistent with the gene-content variation revealed by the PAV analysis, although the absence of a detectable syntenic relationship does not necessarily indicate gene absence.

Overall, the widespread collinearity of *SWEET* genes across the 19 wheat accessions revealed a highly conserved genomic framework accompanied by localized variation among individual genomes.

### 3.6 Selection pressure on wheat *SWEET* genes

Pairwise Ka/Ks analysis was performed among orthologous *SWEET* genes within homologous groups across the 19 wheat accessions to evaluate the selective pressures acting on the wheat *SWEET* gene family (**Figure 6**; **Supplementary Table 9**). After excluding gene pairs with Ks < 0.001 or Ks > 1.5, 11,221 valid pairwise comparisons from 89 *TaSWEET* groups were retained. Of these, 9,470 (84.4%) had Ka/Ks ratios < 1, whereas 1,751 (15.6%) had ratios > 1. Ka/Ks distributions were predominantly concentrated below 1, indicating that purifying selection predominated among the analyzed orthologous pairs. At the gene-group level, mean Ka/Ks ratios ranged from 0.208 to 0.991 and were below 1 for all 89 *TaSWEET* groups. Individual orthologous comparisons nevertheless showed considerable variation, with some Ka/Ks ratios exceeding 1 and the maximum value reaching 2.137. These results indicate that although purifying selection was predominant, selective constraints varied among individual orthologous gene pairs.

**Figure 6:**
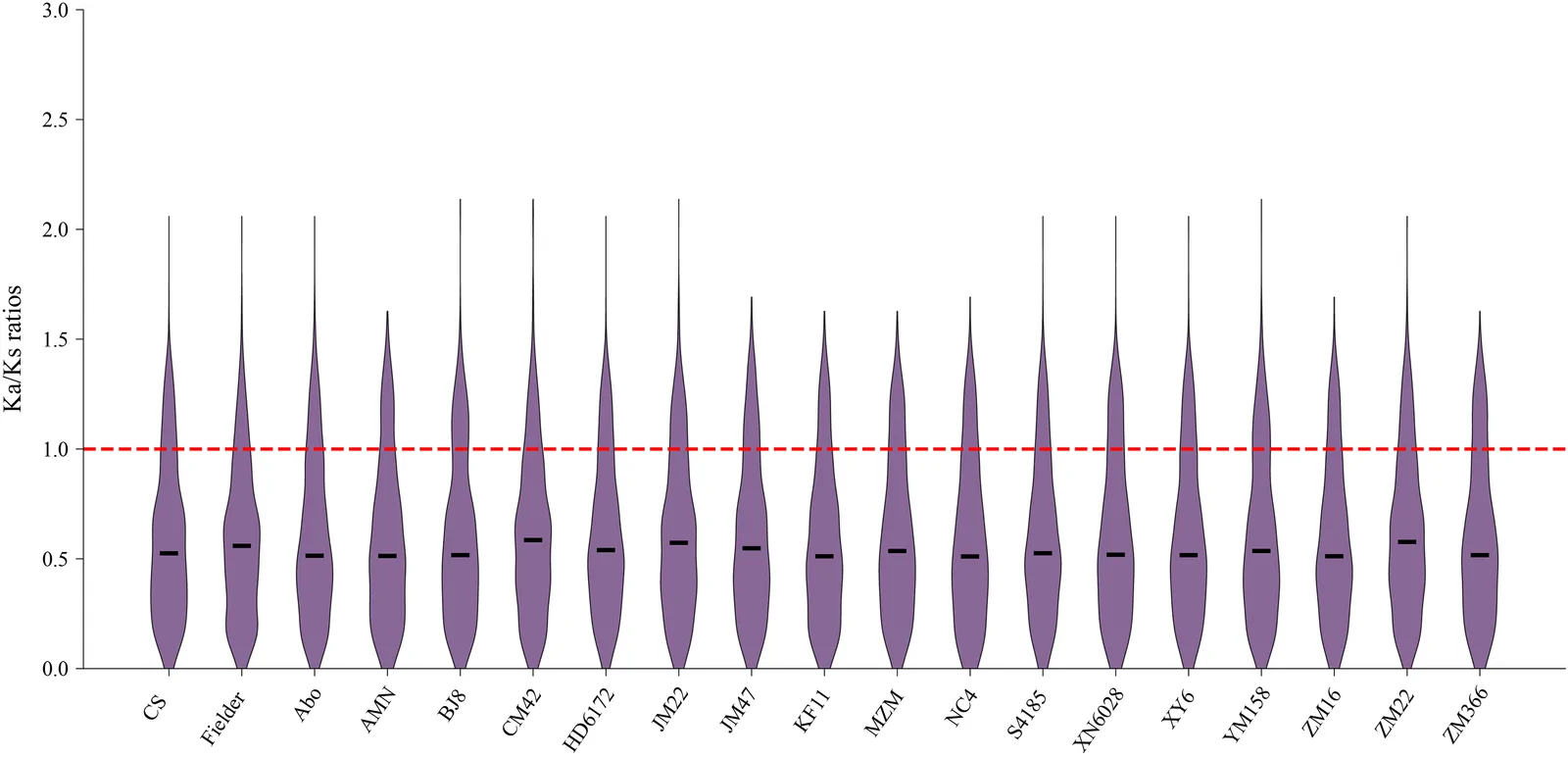
Distribution of Ka/Ks ratios of pairwise *SWEET* orthologs across 19 wheat accessions. Violin plots show the distributions of Ka/Ks ratios calculated for pairwise *SWEET* orthologs across the 19 wheat accessions. Each violin represents the distribution of Ka/Ks ratios for one accession based on pairwise ortholog comparisons with the other wheat accessions. The horizontal dashed line indicates Ka/Ks = 1. Ka/Ks ratios < 1, = 1, and > 1 are generally indicative of purifying selection, neutral evolution, and positive selection, respectively.

Overall, these results indicate that purifying selection has been the predominant selective regime acting on conserved *SWEET* orthologs across the 19 wheat accessions, whereas a smaller proportion of orthologous comparisons showed elevated Ka/Ks ratios > 1, indicating heterogeneous evolutionary constraints among individual gene pairs.

### 3.7 Comparative synteny of *SWEET* genes between wheat, rice, and *Arabidopsis*

Comparative synteny analyses were performed to examine the conservation of *SWEET* genes between CS and *O. sativa*, and between CS and *A. thaliana* (**Figure 7**). *TaSWEET78* and *TaSWEET83* showed conserved orthologous relationships with *AtSWEET5*. In addition, *TaSWEET23*, *TaSWEET24*, *TaSWEET25*, *TaSWEET26*, *TaSWEET27*, *TaSWEET28*, *TaSWEET30*, *TaSWEET31*, *TaSWEET33*, *TaSWEET37*, *TaSWEET40*, *TaSWEET43*, *TaSWEET69*, *TaSWEET78*, and *TaSWEET83* showed syntenic associations with rice *SWEET* genes. *TaSWEET78* and *TaSWEET83* were conserved in both comparisons. Overall, *SWEET*-associated syntenic relationships were more extensive between wheat and rice than between wheat and *Arabidopsis*.

**Figure 7:**
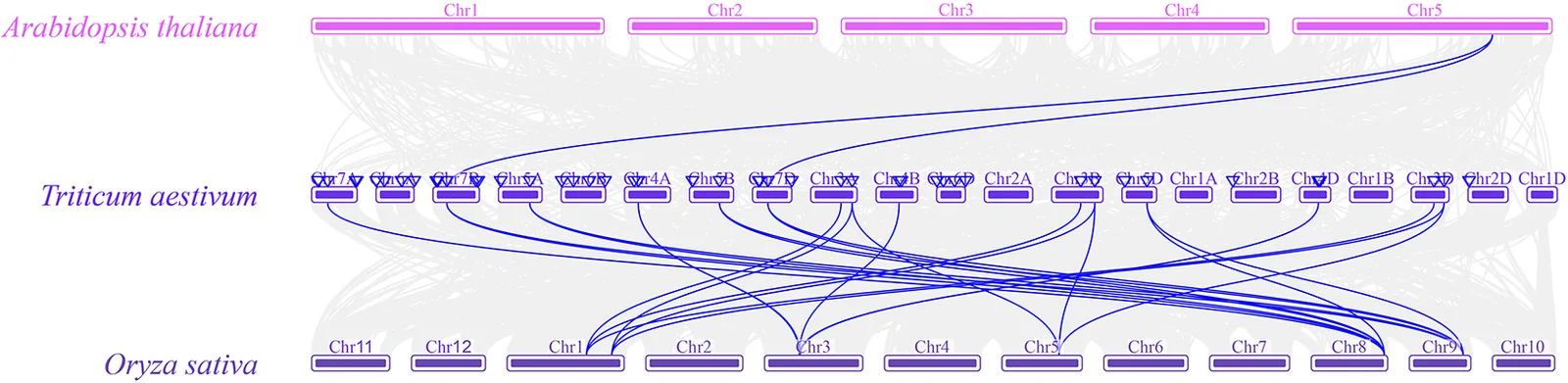
Comparative synteny of *SWEET* genes among *Triticum aestivum*, *Arabidopsis thaliana*, and *Oryza sativa*. Chromosomes of *A. thaliana*, *T. aestivum*, and *O. sativa* are shown from top to bottom. Gray lines in the background represent genome-wide collinear relationships between species, whereas blue lines highlight syntenic relationships involving *SWEET* genes.

### 3.8 Cis-regulatory elements in the promoters of wheat *SWEET* genes

Promoter analysis identified 51 types of putative cis-regulatory elements in the 2-kb upstream regions of *SWEET* genes across the 19 wheat accessions (**Figure 8**; **Supplementary Table 10**). These elements were broadly classified into three functional categories: environmental- and light-responsive elements, hormone-responsive elements, and development- and growth-related elements. Environmental- and light-responsive elements were the most abundant category, followed by hormone-responsive elements, whereas development- and growth-related elements were comparatively less abundant.

**Figure 8:**
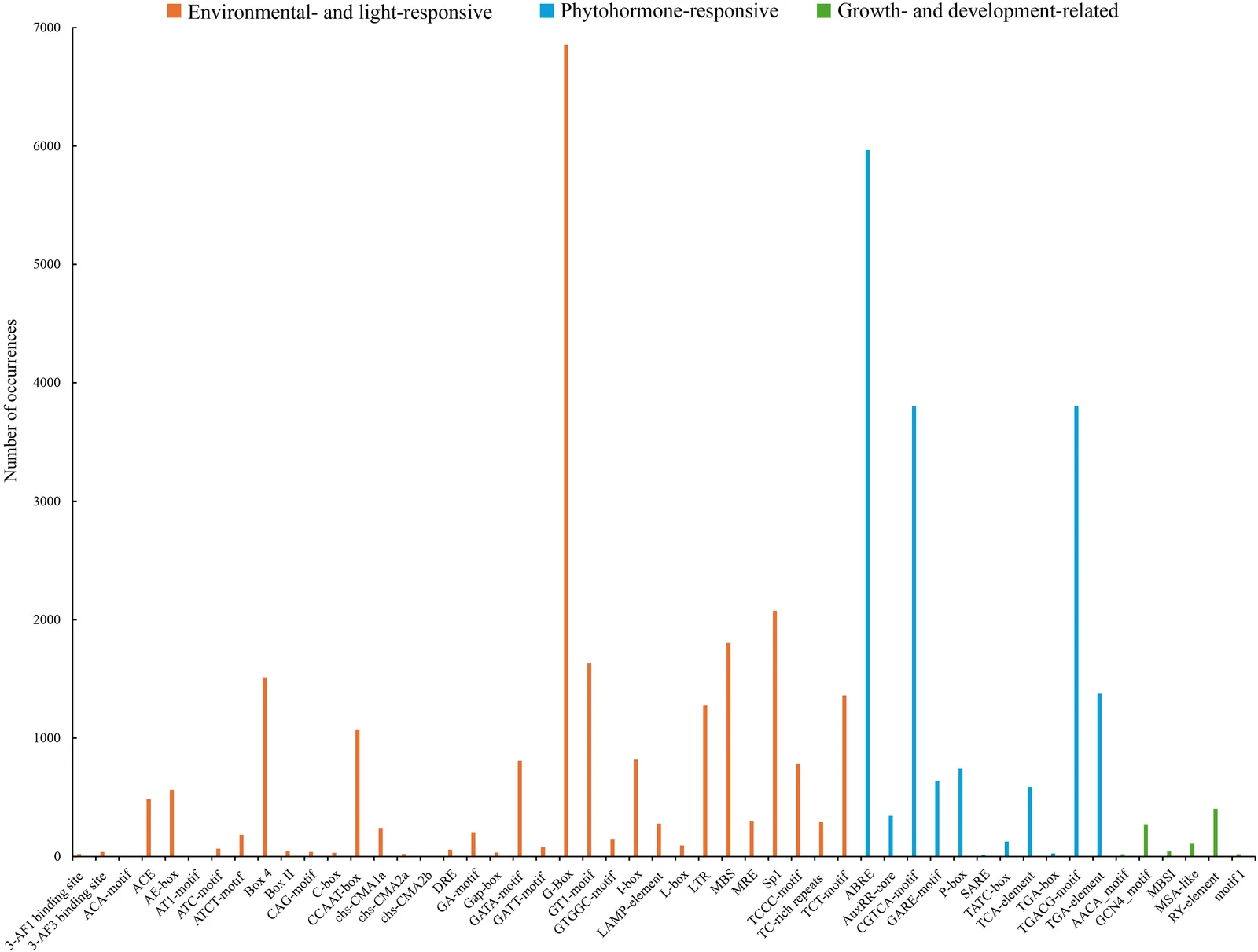
Distribution of predicted cis-regulatory elements in the 2-kb upstream promoter regions of wheat *SWEET* genes across 19 accessions. A total of 51 types of putative cis-regulatory elements were identified and grouped into three broad functional categories for visualization: environmental- and light-responsive, phytohormone-responsive, and growth- and development-related elements. The x-axis indicates cis-element types, and the y-axis indicates the total number of occurrences across the analyzed *SWEET* promoters.

Among the environmental- and light-responsive elements, light-responsive elements were particularly abundant, while elements associated with drought, low-temperature, dehydration, salt, and defense/stress responses were also detected. Hormone-responsive elements were associated with several major phytohormone pathways, including abscisic acid, methyl jasmonate, auxin, gibberellin, and salicylic acid. In addition, several cis-regulatory elements associated with plant growth and developmental processes were identified.

Overall, the predicted cis-regulatory elements showed considerable diversity, with elements associated with environmental, hormonal, and developmental responses represented across the wheat *SWEET* family.

### 3.9 Expression profiles of *TaSWEET* genes across different tissues

RNA-seq data from multiple tissues of CS revealed distinct tissue-associated expression patterns among *TaSWEET* genes (**Figure 9A**; **Supplementary Tables 11A**, **12A**). Following row-wise normalization, several Clade II genes including *TaSWEET47*, *TaSWEET54*, and *TaSWEET63*, showed relatively similar expression patterns in roots, stems, leaves, spikes, and grains. In Clade I, *TaSWEET46*, *TaSWEET53*, and *TaSWEET62* also exhibited relatively broad expression patterns, particularly in roots, stems, and grains. In contrast, several Clade III genes exhibited distinct tissue-preferential expression patterns among the examined tissues. *TaSWEET69*, *TaSWEET78*, and *TaSWEET83* displayed preferential expression in grains, whereas *TaSWEET67*, *TaSWEET73*, and *TaSWEET81* showed preferential expression in spikes. Many other family members exhibited tissue-restricted relative expression. Expression patterns also differed among *TaSWEET* genes within the same phylogenetic clade.

**Figure 9:**
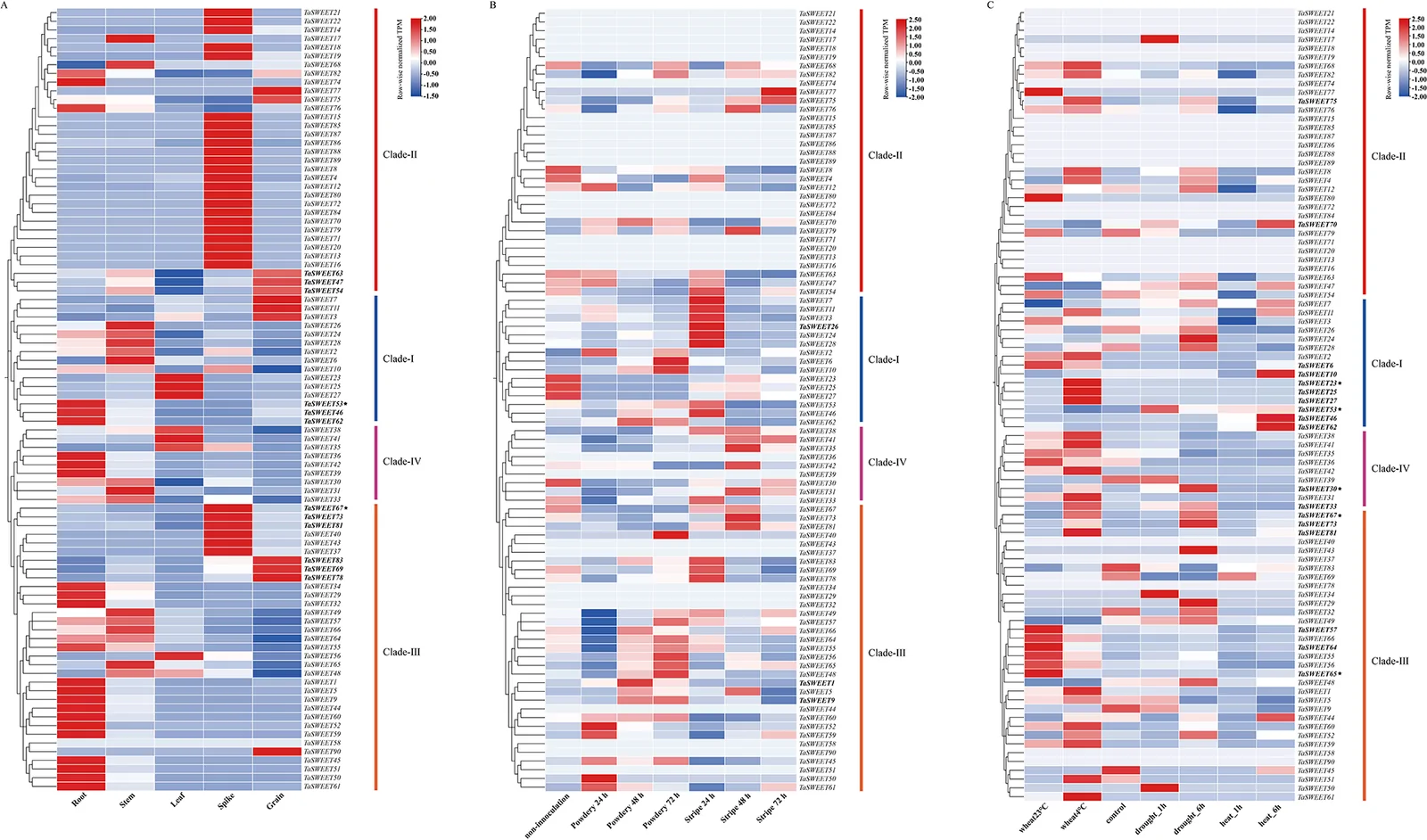
Expression profiles of wheat *TaSWEET* genes across different tissues and environmental conditions. Publicly available RNA-seq datasets were used to examine *TaSWEET* expression patterns. **(A)** Expression profiles across roots, stems, leaves, spikes, and grains. **(B)** Expression profiles following powdery mildew and stripe rust infection at different time points. **(C)** Expression profiles under temperature- and water-related environmental treatments. Transcript abundance was quantified as transcripts per million (TPM), and TPM values were normalized independently for each gene for heatmap visualization. The color scale therefore represents relative expression patterns within individual genes and does not indicate statistically significant differential expression. Phylogenetic clade assignments (Clades I-IV) are indicated on the right. Genes discussed in the text are shown in bold, and qRT-PCR candidate genes are marked with asterisks.

### 3.10 Relationships among PAV, cis-elements, phylogenetic clades, and tissue expression

To examine the relationships among PAV, cis-elements, phylogenetic clades, and tissue expression, we performed a focused analysis of CS *TaSWEET* genes (**Supplementary Table 12A**). The six private genes contained a median of 6.50 selected drought- and cold-related cis-elements (Q1-Q3: 4.75-8.25), compared with 5.00 (4.00-7.00) in the 84 non-private genes (**Supplementary Table 12B**). The difference was not significant (*P* = 0.214), indicating that private genes did not contain significantly fewer selected cis-elements.

The maximum tissue TPM was significantly lower in private genes than in non-private genes, with median values of 0.133 (0.070-0.246) and 1.573 (0.315-3.610), respectively (*P* = 0.008) (**Supplementary Table 12B**). All six private genes had maximum tissue TPM values below 1. Tissue preference was not assigned to these genes because differences among tissues at such low expression levels may not be reliable (**Supplementary Table 12B**).

Clade III contained 33 CS genes, of which 20 (60.6%) were core. In comparison, 24 of the 57 genes (42.1%) in the other clades were core. This difference was not significant (*P* = 0.126). Among expressed genes, 3 of 25 Clade III genes (12.0%) and 4 of 23 genes (17.4%) in the other clades showed grain-preferential expression (*P* = 0.696) (**Supplementary Table 12C**). Thus, Clade III was not significantly enriched in either core or grain-preferential genes in CS.

### 3.11 Expression profiles of *TaSWEET* genes under pathogen infection

RNA-seq analysis revealed distinct pathogen-associated expression patterns of *TaSWEET* genes following infection with powdery mildew (*Bgt*) and stripe rust (*Pst*) (**Figure 9B**; **Supplementary Table 11A**). Following row-wise normalization, *TaSWEET* genes showed different temporal expression patterns across the examined time points, with several genes exhibiting distinct responses to the two fungal pathogens.

Under powdery mildew infection, *TaSWEET1* (Clade III) showed a modest increase in relative expression at 24 h, peaked at 48 h, and decreased at 72 h after inoculation, whereas *TaSWEET26* (Clade I) showed lower relative expression than the control throughout the infection period. *TaSWEET9* (Clade III) showed lower relative expression at 24 h and increased expression at 48 and 72 h.

During stripe rust infection, *TaSWEET26* displayed a transient increase in relative expression at 24 h followed by a decline at 48 and 72 h, whereas *TaSWEET1* remained lower than the non-inoculated level throughout the examined period. *TaSWEET9* also showed a time-dependent response, with decreased expression at 24 h, increased expression at 48 h, and a subsequent decrease at 72 h. Collectively, these results revealed distinct temporal expression patterns of individual *TaSWEET* genes in response to powdery mildew and stripe rust infection.

### 3.12 Expression profiles of *TaSWEET* genes under abiotic stresses

RNA-seq analysis revealed distinct transcriptional responses of *TaSWEET* genes to cold, drought, and heat stresses, with different subsets of genes showing stress-associated expression patterns under each treatment (**Figure 9C**; **Supplementary Table 11A**).

Under cold stress, *TaSWEET23, TaSWEET25*, and *TaSWEET27* (Clade I) showed higher relative expression. *TaSWEET67* and *TaSWEET81* (Clade III) also showed higher relative expression, whereas *TaSWEET57*, *TaSWEET64*, and *TaSWEET65* (Clade III) showed lower relative expression. Under drought stress, *TaSWEET30* and *TaSWEET33* (Clade IV), and *TaSWEET67* (Clade III) maintained higher relative expression during treatment. *TaSWEET53* (Clade I) showed higher relative expression at the early stage, followed by a gradual decrease while remaining above the control level. Under heat stress, *TaSWEET46* and *TaSWEET62* (Clade I) and *TaSWEET73* (Clade III) showed increasing relative expression from 1 to 6 h. *TaSWEET6* and *TaSWEET10* (Clade I), *TaSWEET70*, and *TaSWEET75* (Clade II) showed lower relative expression at 1 h and increased expression at 6 h. *TaSWEET81* (Clade III) changed little at 1 h but showed higher relative expression at 6 h.

Notably, *TaSWEET73* (Clade III) and *TaSWEET75* (Clade II) showed increased expression under cold and drought stress and higher expression at 6 h under heat stress, although their early heat responses differed. Collectively, these results revealed distinct and partially overlapping transcriptional responses of *TaSWEET* genes to cold, drought, and heat stresses.

### 3.13 qRT-PCR analysis of *TaSWEET* gene expression under cold and PEG-induced osmotic stress

To further examine the stress-responsive expression patterns observed in the RNA-seq analysis, six representative *TaSWEET* genes (*TaSWEET23*, *TaSWEET30*, *TaSWEET38*, *TaSWEET53*, *TaSWEET65*, and *TaSWEET67*) were selected for qRT-PCR analysis under cold and PEG-induced osmotic stress (**Figure 10**). Analysis of the 2-kb upstream regions showed that all six genes contained both ABRE and MBS elements, while *TaSWEET23*, *TaSWEET30*, and *TaSWEET38* additionally contained LTR elements; no DRE elements were detected in the 2-kb upstream regions of these six genes (**Supplementary Table 13**).

**Figure 10:**
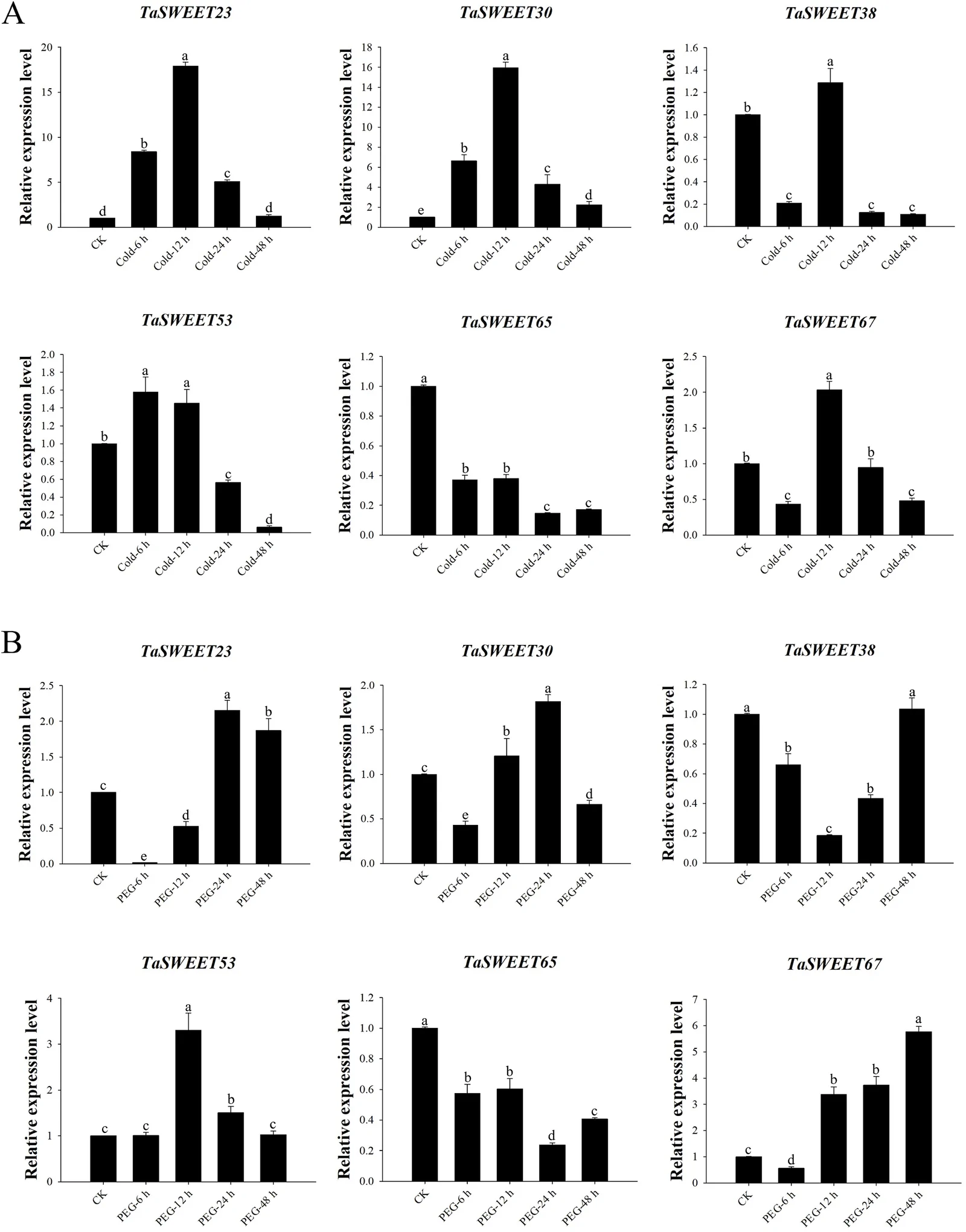
Relative expression profiles of six selected *TaSWEET* genes under cold and PEG-induced osmotic stress. Relative transcript levels of *TaSWEET23*, *TaSWEET30*, *TaSWEET38*, *TaSWEET53*, *TaSWEET65*, and *TaSWEET67* were determined by qRT-PCR under **(A)** cold treatment at 4 °C and **(B)** osmotic stress induced by 20% (w/v) PEG 6000. Samples were collected at 0 h (CK), 6 h, 12 h, 24 h, and 48 h. Relative expression levels were calculated using the 2^^−ΔΔCt^ method with *TaACTIN* as the reference gene and the 0 h samples as the baseline. Values represent means ± SD from three biological replicates. Different lowercase letters indicate significant differences among time points for each gene (P < 0.05; one-way ANOVA followed by Duncan’s multiple range test).

Under cold stress, all six genes exhibited significant temporal changes in transcript abundance (**Figure 10A**). *TaSWEET23* and *TaSWEET30* showed pronounced increases during the early stage of treatment, reaching their highest transcript levels at 12 h, followed by substantial declines at 24 and 48 h. In contrast, *TaSWEET38* was strongly repressed at 6 h, increased at 12 h, and decreased again at 24 h and 48 h. *TaSWEET65* showed sustained repression, reaching its lowest level at 24 h before partially recovering at 48 h. *TaSWEET53* reached its highest transcript level at 6 h and subsequently declined, whereas *TaSWEET67* initially decreased at 6 h, increased markedly at 12 h, and then declined at later time points.

Under PEG-induced osmotic stress, the six genes exhibited distinct temporal expression patterns (**Figure 10B**). *TaSWEET23* and *TaSWEET38* were strongly repressed during the early stages of treatment and subsequently recovered, with transcript levels at 48 h approaching or exceeding their initial levels. *TaSWEET30* decreased at 6 h, increased at 12 and 24 h, and declined again at 48 h. *TaSWEET53* showed a transient increase at 12 h followed by a decline toward the control level. *TaSWEET65* showed an overall decline, reaching its lowest transcript level at 24 h before partially recovering at 48 h. In contrast, *TaSWEET67* was strongly repressed at 6 h but subsequently increased progressively, reaching its highest transcript level at 48 h. Similar overall expression patterns were obtained when *TaGAPDH* was used as an additional reference gene, although the magnitude of some responses differed (**Supplementary Figure 4**, **Supplementary Table 14**).

Although all six genes contained ABRE and MBS elements, their expression patterns under PEG treatment differed markedly. Similarly, the three LTR-containing genes (*TaSWEET23*, *TaSWEET30*, and *TaSWEET38*) showed contrasting responses to cold stress, indicating no simple correspondence between the presence of these cis-elements and temporal expression patterns. Overall, the qRT-PCR results obtained with *TaACTIN* and *TaGAPDH* showed distinct temporal transcriptional responses of the six selected *TaSWEET* genes to cold and PEG-induced osmotic stress.

### 3.14 Effects of *TaSWEET47* overexpression on grain traits

*TaSWEET47* showed the highest grain transcript abundance among the 90 CS *TaSWEET* genes in the RNA-seq dataset (**Supplementary Table 11A**). Analysis of the Longchun35 carpel RNA-seq dataset (Liu et al., 2025) showed that *TaSWEET47* expression increased at heading and peaked at anthesis. After declining at 5 days post-anthesis (DPA), expression increased again at 10–15 DPA and fluctuated during later stages (**Supplementary Table 11C**, **Supplementary Figure 3**). To investigate the potential role of *TaSWEET47* in grain development, two independent *TaSWEET47*-overexpression lines, OE-1 and OE-2, in the Fielder background were selected for further characterization. Positive transgenic plants were confirmed by PCR, and qRT-PCR analysis at 25 days after anthesis showed that *TaSWEET47* transcript levels were significantly higher in both OE-1 and OE-2 than in Fielder (**Figure 11A**, **Supplementary Table 15A**). *TaACTIN* Ct values were similar among Fielder, OE-1, and OE-2, with mean values of 25.51, 25.43, and 25.53, respectively (**Supplementary Table 15A**).

**Figure 11:**
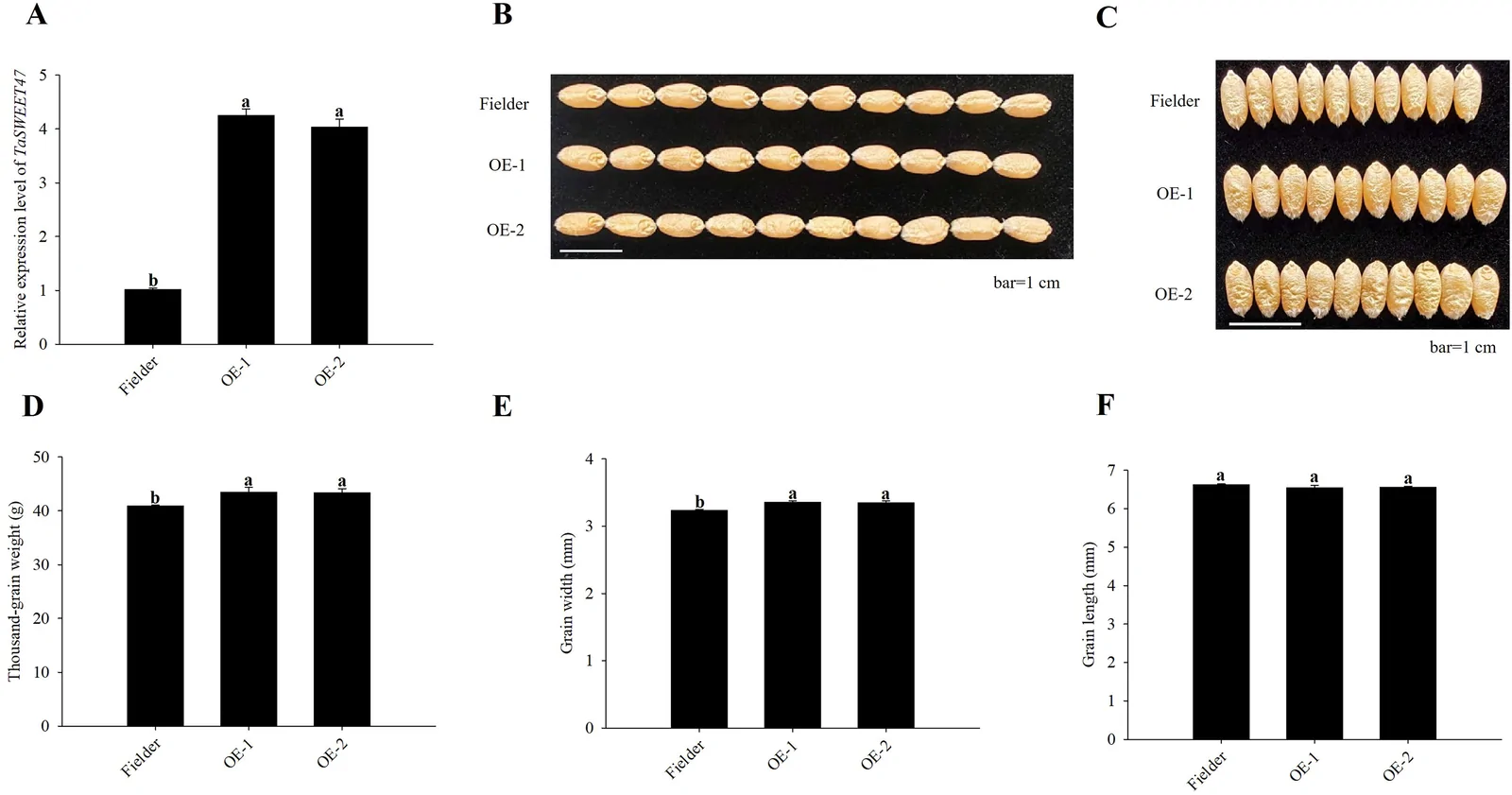
Effects of *TaSWEET47* overexpression on wheat grain-related traits. **(A)** Relative expression levels of *TaSWEET47* in Fielder and two independent T2 overexpression lines (OE-1 and OE-2) at 25 days after anthesis. **(B, C)** Representative mature grain phenotypes of Fielder, OE-1, and OE-2. Scale bars in **(B)** and **(C)** = 1 cm. **(D)** Thousand-grain weight. **(E)** Grain width. **(F)** Grain length. Values are presented as means ± SD. Different lowercase letters indicate significant differences among genotypes (P < 0.05; one-way ANOVA followed by Duncan's multiple range test).

At maturity, both *TaSWEET47*-overexpression lines showed broader grains than Fielder (**Figures 11B, C**). Quantitative analysis showed that thousand-grain weight was significantly increased in both OE-1 and OE-2 compared with Fielder (**Figure 11D**). Grain width was also significantly increased in both overexpression lines (**Figure 11E**), whereas no significant difference in grain length was detected between the overexpression lines and Fielder (**Figure 11F**; **Supplementary Table 15**).

In addition, the starch content of mature grains was significantly higher in both overexpression lines than in Fielder. The starch contents were 53.0 ± 0.4 g/100 g in Fielder, 60.5 ± 0.4 and 55.3 ± 0.5 g/100 g in OE-1 and OE-2, respectively (**Supplementary Figure 5**, **Supplementary Table 15E**).

Collectively, these results indicate that overexpression of *TaSWEET47* is associated with increased grain width, thousand-grain weight, and starch content, without significantly affecting grain length under the conditions examined.

## 4 Discussion

Sugars are not only primary products of photosynthesis but also central metabolites and signaling molecules that coordinate source-sink relationships, growth, reproduction, and responses to environmental challenges (Chen et al., 2015a). SWEET transporters mediate facilitated sugar transport across cellular membranes and have been implicated in phloem loading and unloading, seed and grain filling, reproductive development, and plant-pathogen interactions (Breia et al., 2021; Ji et al., 2022; Xue et al., 2022). Most previous genome-wide studies of plant *SWEET* families have relied on a single reference genome, an approach that cannot fully represent intraspecific gene-content variation. By integrating 19 hexaploid wheat genome assemblies, the present study extends SWEET-family characterization from a single-reference framework to the pan-genome level and reveals extensive conservation accompanied by gene-content variation, coding-sequence conservation, regulatory diversification, and functional differentiation within the wheat *SWEET* family.

### 4.1 A conserved *SWEET* framework accompanied by pan-genomic gene-content variation

A major feature of the wheat *SWEET* family is the coexistence of highly conserved and variable genomic components. Core and soft-core homologous groups together accounted for 83.5% of the 1,594 identified *SWEET* genes, whereas dispensable and private groups accounted for the remaining 16.5%. Thus, most *SWEET* groups were retained across the 19 accessions, but a single reference genome did not contain the complete gene repertoire identified across the wheat pan-genome. This pattern is consistent with the extensive gene-content and structural variation revealed by previous wheat pan-genome analyses (Walkowiak et al., 2020).

Eleven *SWEET* genes were located on unanchored or non-standard genomic sequences. Differences in assembly completeness, chromosome anchoring, and annotation quality among accessions may contribute to this observation. These genes were retained in the *SWEET* gene set but excluded from chromosome-level analyses. Their unresolved chromosome assignments should not be interpreted as evidence of accession-specific chromosomal rearrangements.

Private *SWEET* genes were not uniformly distributed across chromosomes, with chromosome 6B containing the largest number, followed by 2B and 1B. This pattern may be associated with regional genomic variation among wheat accessions, as extensive structural rearrangements and gene-content variation have been reported across wheat genomes (Walkowiak et al., 2020; Jiao et al., 2025). However, the present analysis did not directly characterize the structural mechanisms responsible for the origin of individual private genes. Their higher numbers on particular chromosomes should therefore be interpreted as a genomic distribution pattern rather than as direct evidence for a specific mechanism such as tandem duplication or transposon-mediated insertion.

To further explore whether *SWEET* gene-content composition varied across wheat breeding periods, 17 Chinese accessions were grouped into three breeding periods. The total number of *SWEET* genes and the numbers of core, soft-core, dispensable, private, and variable genes did not differ significantly among the three groups. These observations indicate that the overall *SWEET* gene-family composition remained highly stable among the accessions representing the three breeding periods examined. Because the number of accessions was limited and the three groups were unbalanced, this result should not be interpreted as evidence that modern breeding directly increased or decreased *SWEET* gene content.

The biological significance of the variable *SWEET* component therefore remains to be established. Private and dispensable genes may contribute to accession-specific variation, but their presence or absence alone is insufficient to infer phenotypic or adaptive effects. Larger population-scale pan-genomes incorporating well-defined pedigrees, breeding histories, ecological origins, and phenotypic information will be required to determine whether particular *SWEET* PAVs show consistent patterns of retention or loss during wheat improvement.

### 4.2 Evolutionary conservation and diversification of the wheat *SWEET* family

Phylogenetic analysis using *Arabidopsis* and rice SWEET proteins as references recovered the four classical clades of the *SWEET* family. Most wheat SWEET proteins could be assigned to these clades according to their phylogenetic positions relative to the reference proteins, with Clades II and III containing more members than Clades I and IV. A small set of highly divergent wheat proteins showed less straightforward placement relative to the reference-defined clades, indicating sequence diversification among individual members of the wheat *SWEET* family. Previous functional studies have shown that Clade III SWEETs frequently transport sucrose and participate in source-sink allocation, reproductive development, seed filling, and plant-pathogen interactions, whereas members of Clades I and II are more commonly associated with hexose transport and Clade IV members with vacuolar sugar transport and intracellular sugar homeostasis (Chen et al., 2010, 2012, 2015b; Chardon et al., 2013; Klemens et al., 2013; Lin et al., 2014; Kanno et al., 2016). The large representation of Clades II and III in hexaploid wheat is therefore noteworthy, although clade membership alone does not establish substrate specificity or biological function for individual wheat proteins.

Clade size alone also does not demonstrate lineage-specific adaptive expansion or differences in evolutionary rate. Bread wheat is an allohexaploid and consequently retains homoeologous copies of many genes in the A, B, and D subgenomes. The extensive A-B, A-D, and B-D homoeologous collinearity detected in this study, together with the high level of inter-accession synteny, indicates substantial retention of conserved *SWEET* genomic relationships following wheat polyploid evolution. Comparative synteny with rice was considerably more extensive than with *Arabidopsis*, consistent with the closer evolutionary relationship between wheat and rice as monocots. These observations are compatible with a contribution of polyploidization and subsequent gene retention to the large *SWEET* complement of hexaploid wheat, but they do not quantify the contribution of individual polyploidization events. Direct comparisons with diploid and tetraploid wheat relatives will be required to reconstruct *SWEET* copy-number changes during wheat polyploid evolution.

The Ka/Ks analysis provides an additional perspective on the evolutionary history of conserved *SWEET* orthologs. Most pairwise ortholog comparisons had Ka/Ks ratios below 1, indicating that purifying selection has predominated during the evolution of conserved *SWEET* coding sequences. A smaller subset of comparisons showed Ka/Ks values above 1, suggesting heterogeneous evolutionary constraints among individual orthologous pairs. Together with the extensive inter-accession synteny and homoeologous collinearity, these results indicate substantial evolutionary constraint on SWEET protein-coding sequences, together with heterogeneous evolutionary constraints among a subset of orthologous pairs. Because pairwise Ka/Ks estimates alone are insufficient to demonstrate site-specific positive selection, elevated ratios in individual comparisons should be interpreted cautiously and require further evaluation using codon-based evolutionary models.

Structural characteristics further support the coexistence of conservation and diversification. Motif analysis across all 19 accessions revealed broadly clade-associated conservation of motif composition together with variation among individual members, while domain and exon-intron comparisons between CS and Fielder showed generally conserved *SWEET* architectures. In this context, evolutionary constraint refers to preservation of major structural features likely required for transporter function rather than an absence of sequence evolution. Overall, the wheat *SWEET* family retained a broadly conserved structural framework while showing variation in gene content and motif composition among individual members.

### 4.3 Cis-regulatory elements and heterogeneous expression patterns

The two prediction methods gave different subcellular localization assignments for some SWEET proteins. These differences may reflect variation in their algorithms, training datasets, and sequence features used for prediction. The assignments should therefore be regarded as provisional rather than experimental evidence of localization. For TaSWEET47, the fluorescence signal overlapped with the plasma membrane marker SNAP::R, providing experimental support for its predominant plasma membrane localization.

Analysis of the 2-kb upstream regions revealed a complex cis-regulatory landscape within the wheat *SWEET* family. G-Box elements were particularly abundant, while ABRE and the MeJA-responsive CGTCA- and TGACG-motifs were also highly represented. ABREs are well-established ABA-responsive cis-elements, whereas CGTCA- and TGACG-motifs have been associated with MeJA responsiveness (Guiltinan et al., 1990; Uno et al., 2000; Wang et al., 2011). Elements associated with low-temperature, dehydration/osmotic, defense, and developmental responses were also detected in *SWEET* promoters. These findings indicate that wheat *SWEET* genes possess diverse putative regulatory architectures associated with environmental, hormonal, and developmental responses. Nevertheless, the presence or abundance of a predicted cis-regulatory motif cannot by itself establish functional regulation by the corresponding stimulus (Ezer et al., 2017).

Integration of promoter predictions with expression data provides a more informative view of this regulatory diversity. Across tissues, pathogen infection, and abiotic treatments, *TaSWEET* genes displayed heterogeneous expression profiles, including among members of the same phylogenetic clade. Genes containing similar stress-associated cis-elements also showed different temporal responses, indicating that the presence of individual predicted motifs does not necessarily correspond to a common transcriptional pattern. Such differences may reflect variation in cis-regulatory sequence context, motif combinations, and trans-acting regulatory factors (Schmitz et al., 2022).

The pan-genome comparisons further suggest that gene presence/absence and regulatory variation represent related but distinct dimensions of *SWEET* diversity. Private and non-private genes did not differ significantly in the number of the selected stress-related cis-elements, suggesting that accession specificity was not simply associated with reduced abundance of these predicted motifs. Private genes nevertheless showed lower tissue expression than non-private genes, indicating that differences in transcriptional behavior cannot be explained solely by the number of examined cis-elements. Because the present analysis focused on predicted promoter motifs rather than accession-resolved promoter SNPs, InDels, transposable-element insertions, or other structural variants, the molecular basis of this association remains unresolved. Integration of promoter haplotypes with matched accession-specific transcriptomes will be necessary to distinguish the contributions of PAV and sequence-level regulatory variation.

### 4.4 Tissue-associated expression and the role of *TaSWEET47* in grain-related traits

Tissue transcriptome analysis revealed marked differentiation in the relative expression patterns of wheat *SWEET* genes. Several genes showed relatively higher expression in reproductive tissues, with *TaSWEET69*, *TaSWEET78*, and *TaSWEET83* showing particularly strong grain-associated expression patterns. Such expression is consistent with the known involvement of several *SWEET* transporters in sucrose allocation and seed development. In rice, *OsSWEET11* contributes to sucrose transport during seed development, and disruption of SWEET-mediated sugar transport impairs grain filling (Ma et al., 2017). Recent functional analysis in wheat further demonstrated that *TaSWEET11* is predominantly expressed in developing grains and participates in sucrose transport and starch accumulation; disruption of *TaSWEET11* impairs grain filling, whereas its overexpression can enhance yield-related traits (Wang et al., 2025). The grain-associated expression of *SWEET11*-related genes detected in the present study supports their prioritization as candidates for grain-related functions. However, grain-preferential expression was not significantly enriched in Clade III, indicating that phylogenetic position alone was not sufficient to predict grain-associated expression.

Functional characterization of *TaSWEET47* provides additional evidence that individual wheat *SWEET* genes can influence grain-related traits. Multiple lines of comparative and transcriptomic evidence support the functional relevance of *TaSWEET47* in grain filling. *TaSWEET47* is the ortholog of rice *OsSWEET4* and maize *ZmSWEET4c*, which are known hexose transporters that mediate sugar translocation into endosperm and control seed filling (Mangini et al., 2025; Sosso et al., 2015). Consistently, *TaSWEET47* showed the highest grain transcript abundance among all 90 *TaSWEET* genes in the CS RNA-seq dataset. Public transcriptome data of wheat cultivar Longchun35 further revealed that *TaSWEET47* exhibits dynamic expression peaking at anthesis and remaining high during the critical grain-filling stage (10–15 days post-anthesis).

Two independent *TaSWEET47*-overexpression lines exhibited significantly increased grain width and thousand-grain weight compared with Fielder, whereas grain length was not significantly altered. Starch content was also higher in both overexpression lines than Fielder. The concordant phenotypes of the two independent lines support an association between elevated *TaSWEET47* expression and increased grain width and grain weight. These findings extend the pan-genomic and transcriptomic analyses by providing preliminary functional screening evidence that altered *TaSWEET47* expression can affect grain-related traits.

The *TaSWEET47* phenotype should nevertheless be interpreted within the broader complexity of source-sink regulation. *SWEET* transporters alter the spatial distribution and availability of sugars rather than simply increasing total carbohydrate production, and changes in transporter abundance may therefore affect specific components of grain development or filling (Chen et al., 2012; Breia et al., 2021). Although the higher starch content was consistent with the increased grain weight in the overexpression lines, it remains unclear whether this resulted directly from altered sugar transport, changes in grain-filling rate or duration, or other downstream processes. Measurements of sugar transport, soluble carbohydrate, spatial expression, and grain-filling dynamics will be required to establish the physiological mechanism underlying this phenotype. Moreover, increased thousand-grain weight should not be equated with increased whole-plant or field yield without direct yield measurements.

### 4.5 *SWEET* genes exhibit diverse transcriptional responses to biotic and abiotic stresses

SWEET transporters are positioned at the interface between primary metabolism and environmental responses. During powdery mildew and stripe rust infection, several *TaSWEET* genes exhibited temporal and pathogen-dependent expression patterns. These responses were not restricted to a single phylogenetic group, indicating that pathogen-associated transcriptional responses varied among individual *SWEET* genes rather than representing a uniform property of an entire clade.

*TaSWEET9* showed a dynamic temporal response during fungal infection. Notably, *TaSWEET9* corresponds to *TaSWEET14d* (*TraesCS1D02G052700*), which has recently been functionally implicated in the wheat-*Puccinia striiformis* interaction. Previous work showed that *Pst* can induce *TaSWEET14d* expression and exploit host sugar transport to facilitate infection, providing independent evidence that this *SWEET* member participates in the wheat-stripe rust interaction (Zheng et al., 2025). The expression response detected in the present analysis is therefore consistent with an experimentally supported pathogen-associated role of *TaSWEET14d*. Nevertheless, transcript abundance alone cannot establish whether other responsive *SWEET* genes promote resistance, susceptibility, or pathogen nutrition.

Abiotic-stress transcriptomes likewise revealed heterogeneous responses to cold, drought, and heat treatments. Rather than identifying a uniformly stress-responsive clade, the data showed gene-specific and time-dependent expression patterns across the family. *TaSWEET67* was among the genes showing responses under multiple treatments and therefore represents a useful candidate for further functional analysis. By contrast, other genes exhibited more condition-dependent profiles, emphasizing that relative expression changes in public RNA-seq heatmaps should be interpreted as transcriptional patterns rather than formal evidence of statistically significant differential expression.

Six genes were selected for qRT-PCR based on their different expression patterns in public cold and drought RNA-seq datasets (**Supplementary Table 11A**). Relative to the corresponding controls, *TaSWEET23* showed higher expression under cold treatment but lower expression under drought. *TaSWEET30* and *TaSWEET67* showed higher expression under cold and at both drought time points, with a larger increase at 6 h than at 1 h. *TaSWEET38* showed a modest increase under cold, remained close to the control at 1 h under drought, and decreased at 6 h. Both *TaSWEET53* and *TaSWEET65* showed lower expression under cold. Under drought, *TaSWEET53* increased at 1 h and then decreased at 6 h, although its expression remained above the control. *TaSWEET65* remained close to the control at 1 h and increased at 6 h. These genes were selected to represent contrasting response directions and temporal patterns in the RNA-seq datasets. Their expression was subsequently examined by qRT-PCR under cold and PEG-induced osmotic stress.

The qRT-PCR experiments suggested distinct temporal responses of six selected genes under cold and PEG-induced osmotic stress. Some genes exhibited transient increases, others sustained decreases, and *TaSWEET67* showed a delayed increase during PEG treatment. These contrasting kinetics suggest that individual *SWEET* genes may participate at different stages of the transcriptional response to environmental stress. The selected genes also contained stress-associated promoter elements, including ABRE, MBS, and/or LTR elements. However, genes containing the same elements did not necessarily show similar temporal expression patterns, indicating that the presence of individual predicted cis-elements alone was not sufficient to account for the observed expression responses. Direct causal relationships between individual cis-elements and temporal transcriptional responses remain untested. Promoter deletion, reporter assays, and cis-element mutagenesis will be required to determine whether these motifs directly contribute to the observed stress-associated expression patterns.

The qRT-PCR revealed dynamic responses of the selected *TaSWEET* genes to cold and PEG-induced osmotic stress. Some trends were consistent with the RNA-seq data, whereas others differed across time points, which may reflect differences in experimental conditions, sampling strategies, and detection methods. To further check the qRT-PCR results, *TaGAPDH* was used as an additional reference gene. The expression patterns obtained with *TaACTIN* and *TaGAPDH* were generally similar, although the extent of some expression changes differed between the two reference genes. These results support the main stress-responsive expression patterns observed in the selected genes.

### 4.6 Implications for wheat improvement

The integration of pan-genomic variation, expression profiling, and functional evidence identifies several potential routes for exploiting SWEET diversity in wheat improvement. For grain-related traits, grain-associated *SWEET11*-related genes and experimentally characterized *TaSWEET47* represent distinct candidate resources. Natural promoter and coding haplotypes of these genes could be investigated across larger wheat diversity panels and tested for associations with grain size, grain filling, thousand-grain weight, and other yield components. If trait-associated natural alleles are identified, they could be further evaluated for marker development and breeding applications. For genes with experimentally validated effects, targeted modification of regulatory regions may provide an alternative strategy for fine-tuning gene expression.

Stress-associated *SWEET* genes require particularly careful evaluation because altered sugar transport can have context-dependent consequences. Increased sugar availability may benefit sink development under some conditions while potentially increasing nutrient availability to pathogens (Chen et al., 2010; Bezrutczyk et al., 2018; Breia et al., 2021). Stress-responsive candidates such as *TaSWEET67* require functional validation before their potential breeding value can be assessed. Loss- and gain-of-function materials and promoter editing should be evaluated under controlled stress conditions before breeding applications are considered. Pan-genome haplotype analysis combined with multi-environment phenotyping could subsequently determine whether naturally occurring *SWEET* variants confer favorable combinations of grain-related performance and stress adaptation.

More broadly, the pan-genome analysis provides a broader basis for prioritizing candidate *SWEET* genes than a single-reference approach (Montenegro et al., 2017; Walkowiak et al., 2020; Bayer et al., 2022). PAV identifies family members not represented uniformly across wheat genomes, cis-element analyses reveal potential regulatory diversity, expression profiles prioritize condition-associated candidates, and functional genetics can establish causal relationships. Integrating these levels of evidence should provide a stronger basis for candidate prioritization than relying on sequence conservation or expression patterns alone.

### 4.7 Limitations and future perspectives

Several limitations of the present study should be considered. First, although 19 high-quality hexaploid wheat genomes substantially expand representation of *SWEET* diversity beyond a single reference genome, they represent only a fraction of global wheat diversity (Walkowiak et al., 2020; Bayer et al., 2022). The comparison among three breeding periods suggested overall stability of *SWEET* gene-family composition with no significant difference detected in any PAV component among the periods, however, only 17 Chinese accessions were included and the groups were unequal in size. Larger and more balanced panels with detailed pedigrees will therefore be required to determine whether particular *SWEET* PAVs have been affected by breeding selection.

Second, the promoter analysis focused on predicted cis-regulatory motifs and did not systematically characterize accession-specific promoter SNPs, InDels, transposable-element insertions, or other structural variants that could contribute to transcriptional divergence. Third, most expression analyses were based on publicly available transcriptomic datasets generated under different experimental designs. They therefore provide comparative relative-expression patterns rather than uniformly generated statistical evidence of differential expression.

Fourth, phylogenetic classification recovered the four classical *SWEET* clades but also identified a small number of highly divergent wheat proteins whose precise placement relative to the reference-defined groups was less straightforward. Additional phylogenetic analyses incorporating broader grass *SWEET* diversity and complementary evolutionary models may help resolve these members. Finally, functional evidence was obtained for *TaSWEET47* through overexpression analysis, whereas the biological functions of most candidates identified through the pan-genome and expression analyses remain unresolved. Increased grain width, thousand-grain weight, and starch content in *TaSWEET47*-overexpression lines support a grain-related phenotype but do not yet reveal its physiological mechanism or demonstrate increased whole-plant yield.

Future work should therefore integrate larger wheat pan-genomes with population-scale haplotype analysis and multi-environment phenotyping to identify naturally occurring *SWEET* variants associated with agronomic traits. CRISPR/Cas-mediated knockout, promoter editing, and complementary overexpression analyses will be required to establish the functions of prioritized genes and regulatory variants (Liu et al., 2021; Zhou et al., 2023). For grain-associated candidates, measurements of sucrose transport, soluble-sugar distribution, starch accumulation during grain development, grain-filling dynamics, and source-sink carbon allocation will be particularly informative. For stress-associated candidates, genetic and physiological analyses under controlled cold, osmotic, heat, and pathogen treatments should be combined with promoter-reporter and cis-element mutagenesis experiments. Such integrated genetic, molecular, and physiological analyses will help clarify how gene-content and regulatory variation contribute to functional differences among wheat *SWEET* genes and determine which natural or engineered alleles have potential value for wheat improvement.

## 5 Conclusions

This study provides a pan-genome-wide characterization of the *SWEET* gene family across 19 hexaploid wheat accessions and reveals a predominantly conserved genomic framework accompanied by a smaller accession-variable component. Gene copies assigned to the core and soft-core categories together accounted for 83.5% of the 1,594 *SWEET* genes, whereas dispensable and private groups accounted for 16.5%, demonstrating substantial conservation while capturing gene-content variation that cannot be represented by a single reference genome. Comparison of 17 Chinese accessions from three breeding periods further indicated that overall *SWEET* gene-family composition remained highly stable, with no significant differences detected in any PAV component among the periods examined.

Phylogenetic, structural, collinearity, and Ka/Ks analyses revealed extensive evolutionary conservation accompanied by diversification within the wheat *SWEET* family. The four classical SWEET clades were recovered, with Clades II and III containing the largest numbers of wheat members, while a small number of highly divergent proteins showed less straightforward phylogenetic placement. Extensive homoeologous and inter-accession collinearity and the predominance of Ka/Ks ratios below 1 indicate strong conservation of *SWEET* coding sequences. In parallel, PAV, motif variation, promoter cis-regulatory diversity, and heterogeneous tissue- and stress-associated expression patterns demonstrate diversification at genomic and regulatory levels.

The integration of expression profiling and functional analysis identified several candidate genes of particular interest. Grain-associated expression was observed for multiple *SWEET* members, and overexpression of *TaSWEET47* significantly increased grain width, thousand-grain weight, and mature-grain starch content without significantly affecting grain length, providing functional evidence for its association with grain-related traits. Other *SWEET* genes displayed distinct responses to pathogen infection and abiotic treatments, and *TaSWEET67* emerged as a candidate for further investigation of stress-associated functions.

Collectively, these results establish a pan-genomic framework for understanding conservation and diversification of the wheat *SWEET* family and provide candidate genomic resources for future functional studies and molecular breeding. Population-scale haplotype analysis, targeted gene and promoter editing, physiological characterization of sugar transport and source-sink allocation, and multi-environment phenotyping will be required to determine which natural or engineered *SWEET* alleles can be effectively utilized for improvement of wheat grain-related and stress-adaptive traits.

## Data Availability

The genome assemblies and annotation files used in this study were obtained from publicly available databases, including Ensembl Plants for CS, the KOMUGI database for Fielder, and the National Genomics Data Center (NGDC) for the remaining wheat cultivars. Publicly available RNA-seq datasets were obtained from the WheatOmics Gene Expression database. The relevant accession and project identification numbers are included in this article and its **Supplementary Materials**.
