# Supplementary material for: Pan-genome analysis reveals conservation and diversification of the SWEET gene family in wheat
Source: Front Plant Sci. 2026 Oct 16;17:1949812. doi: 10.3389/fpls.2026.1949812 (PMC13719393; doi:10.3389/fpls.2026.1949812)
Supplement: Supplementary file 1 [file DataSheet1.docx]

**Supplementary Figure 1.** Representative qRT-PCR melting curves for six *TaSWEET* genes and the two reference genes *TaACTIN* and *TaGAPDH*. (A) *TaSWEET23*; (B) *TaSWEET30*; (C) *TaSWEET38*; (D) *TaSWEET53*; (E) *TaSWEET65*; (F) *TaSWEET67*; (G) *TaACTIN*; (H) *TaGAPDH*. The x-axis indicates temperature (°C), and the y-axis shows the negative derivative of fluorescence with respect to temperature (−dRFU/dT).

**Supplementary Figure 2.** Subcellular localization of TaSWEET47 in tobacco leaf epidermal cells. Upper row: empty p1300-EGFP vector (Null::G) co-expressed with Ras1::R, a nuclear and membrane marker. Lower row: TaSWEET47-GFP co-expressed with the plasma membrane marker SNAP::R. BF, bright-field image; GFP, green fluorescence; RFP, red fluorescence; Merge, overlay of bright-field and fluorescence images. Scale bars = 20 μm.

**Supplementary Figure 3.** Expression patterns of *TaSWEET47* at different developmental stages in wheat cultivar Longchun35 RNA-seq. GrA, green anther stage; YA, yellow anther stage; TP, tipping stage; HD, heading stage; AN, anthesis stage; 5-35 DPA, 5-35 days post-anthesis. The y-axis represents gene expression level (TPM). Data were retrieved from the publicly available Longchun35 transcriptome dataset reported by Liu et al. (2025).

**Supplementary Figure 4.** Independent qRT-PCR validation of six *TaSWEET* genes using *TaGAPDH* as the reference gene under cold and PEG-induced osmotic stress. Relative transcript levels of *TaSWEET23*, *TaSWEET30*, *TaSWEET38*, *TaSWEET53*, *TaSWEET65*, and *TaSWEET67* were determined by qRT-PCR under (A) cold treatment at 4 °C and (B) osmotic stress induced by 20% (w/v) PEG 6000. Samples were collected at 0 h (CK), 6 h, 12 h, 24 h, and 48 h. Relative expression levels were calculated using the 2^^−ΔΔCt^ method with *TaGAPDH* as the reference gene and the 0 h samples as the baseline. Values represent means ± SD from three biological replicates. Different lowercase letters indicate significant differences among time points for each gene (*P* < 0.05; one-way ANOVA followed by Duncan’s multiple range test).

**Supplementary Figure 5.** Starch content of mature grains in Fielder and *TaSWEET47* overexpression lines. Values are means ± SD of three biological replicates. Different lowercase letters indicate significant differences among genotype (*P* < 0.05; one-way ANOVA followed by Duncan’s multiple range test).
